# COMBImage2: a parallel computational framework for higher-order drug combination analysis that includes automated plate design, matched filter based object counting and temporal data mining

**DOI:** 10.1186/s12859-019-2908-0

**Published:** 2019-06-04

**Authors:** Efthymia Chantzi, Malin Jarvius, Mia Niklasson, Anna Segerman, Mats G. Gustafsson

**Affiliations:** 1 Department of Medical Sciences, Cancer Pharmacology and Computational Medicine, Uppsala University, Uppsala, Sweden; 2 SciLifeLab Drug Discovery and Development, In Vitro Systems Pharmacology Facility, Uppsala University, Uppsala, Sweden; 3 Department of Immunology, Genetics and Pathology, Rudbeck Laboratory, Uppsala University, Uppsala, Sweden

**Keywords:** Label-free time-lapse video microscopy, Automated plate design, Higher-order drug combination analysis, Matched filter, Resampling, Data mining, MapReduce, CUSP9v4, Glioblastoma

## Abstract

**Background:**

Pharmacological treatment of complex diseases using more than two drugs is commonplace in the clinic due to better efficacy, decreased toxicity and reduced risk for developing resistance. However, many of these higher-order treatments have not undergone any detailed preceding in vitro evaluation that could support their therapeutic potential and reveal disease related insights. Despite the increased medical need for discovery and development of higher-order drug combinations, very few reports from systematic large-scale studies along this direction exist. A major reason is lack of computational tools that enable automated design and analysis of exhaustive drug combination experiments, where all possible subsets among a panel of pre-selected drugs have to be evaluated.

**Results:**

Motivated by this, we developed COMBImage2, a parallel computational framework for higher-order drug combination analysis. COMBImage2 goes far beyond its predecessor COMBImage in many different ways. In particular, it offers automated 384-well plate design, as well as quality control that involves resampling statistics and inter-plate analyses. Moreover, it is equipped with a generic matched filter based object counting method that is currently designed for apoptotic-like cells. Furthermore, apart from higher-order synergy analyses, COMBImage2 introduces a novel data mining approach for identifying interesting temporal response patterns and disentangling higher- from lower- and single-drug effects.

COMBImage2 was employed in the context of a small pilot study focused on the CUSP9v4 protocol, which is currently used in the clinic for treatment of recurrent glioblastoma. For the first time, all 246 possible combinations of order 4 or lower of the 9 single drugs consisting the CUSP9v4 cocktail, were evaluated on an in vitro clonal culture of glioma initiating cells.

**Conclusions:**

COMBImage2 is able to automatically design and robustly analyze exhaustive and in general higher-order drug combination experiments. Such a versatile video microscopy oriented framework is likely to enable, guide and accelerate systematic large-scale drug combination studies not only for cancer but also other diseases.

**Electronic supplementary material:**

The online version of this article (10.1186/s12859-019-2908-0) contains supplementary material, which is available to authorized users.

## Background

Pharmacological treatment of complex and/or co-occurring diseases using more than two drug compounds simultaneously is commonplace in the clinic [1, 2]. However, many of these multidrug regimens [3–7] have not been systematically studied using conventional in vitro experiments with respect to their desired therapeutic effects and potential adverse side effects. Moreover, a growing activity in any modern drug discovery and development (DDD) project is in vitro evaluation of novel multidrug treatment candidates [8]. Ideally such in vitro evaluations should not be restricted to the widely employed single endpoint analyses, such as cell viability, but rather provide temporal information about changes relative to untreated controls. In order to meet this need, the previously introduced computational framework, COMBImage [9], was designed for label-free time-lapse video microscopy (TLVM) based analysis of pairwise drug combination experiments. COMBImage has already been successfully used in different ongoing and completed DDD projects but, as presented in more detail below, it still has some obvious limitations. Therefore, we developed COMBImage2, which compared to COMBImage (Table 1), offers refined quality control (QC) procedures that now include resampling statistics and inter-plate analyses as well as:

1. automated design of 384-well plate layouts for drug combination experiments of any order

**Table 1:** Modular comparison of COMBImage2 and COMBImage

Readout	Module	Description	Layout	Analysis	COMBImage2	COMBImage
	COMBO-Pick	automated experimental design	P, PE, E		+	-
TLVM	COMBO-MF	matched filter based object counting	P, PE, E	intra,inter	+	-
	COMBO-C	changes in cell confluence	P	intra	+	+
			P	inter	+	-
			PE, E	intra,inter	+	-
	COMBO-M	changes in cell morphology	P	intra	+	+
			P	inter	+	-
			PE, E	intra,inter	+	-
CVA	COMBO-V	cell viability & synergy analyses	P	intra	+	+
			P	inter	+	-
			PE, E	intra,inter	+	-
TLVM, CVA	COMBO-Mine	temporal data mining	PE, E	intra, inter	+	-

Abbreviations are defined as follows. TLVM: time-lapse video microscopy, CVA: cell viability assay, P: pairwise (only pairs of drugs are evaluated in a checkerboard format), PE: partially exhaustive (particular subsets among a panel of drugs are evaluated), E: exhaustive (all possible subsets among a panel of drugs are evaluated), intra: intra-plate analysis (experiment performed in a single experimental plate), inter: inter-plate analysis (experiment replicated in several plates)
2. matched filter based object counting for quantification of particular cellular objects such as apoptotic-like cells and vesicle formations
3. identification, visualization and characterization of prototypical response behaviors, which are used to disentangle higher- from lower- and single-drug effects.

As also elaborated on below, the potential of COMBImage2 was illustrated in the context of a small pilot study covering 255 treated and 53 untreated experimental wells in quadruplicate; each containing all possible combinations of 9 drugs up to order 4, including single drugs. Although this particular study did not provide any outstanding pharmacological findings, it clearly demonstrates the great potential of COMBImage2 as a generic in vitro DDD tool for automated design and analysis of drug combination experiments of any order and type.

### Limitations of COMBImage and other methods

Despite the novelty of COMBImage compared to other tools [10–12], mainly related to the joint employment of cell viability and label-free temporal quantitative microscopy, it only supports the analysis of drug pairs. This is a substantial limitation given the increased medical need for multidrug (i.e., three or more drugs) therapies, in order to achieve better efficacy, decreased toxicity and reduced risk for drug resistance [1, 2]. Moreover, COMBImage offers automated quantification of temporal changes in cell growth/confluence and morphology between treated and untreated cells. Although this enables temporal detection of either interesting drug induced effects or anomalies, it does not allow for dynamic monitoring of specific (sub-)cellular processes, for example induction of apoptosis. Such a methodological advancement would be very valuable for in vitro drug combination analysis and in silico prediction of promising drug combinations [13, 14].

As exemplified in the remaining part of this subsection, attempts along this direction have been reported, but we are not aware of any that can offer accurate cell/object counting in adherent cell cultures studied in a large-scale 384-well format. The previously introduced detectors, LFAD [15] and LFVD [16], have been successfully used to detect drugs that induce apoptosis and intracellular vesicle formation respectively, in the context of in vitro cancer pharmacology studies. They employ a very similar experimental set up to COMBImage, as they are also able to process phase-contrast images from adherent cell cultures in a 384-well format. However, they cannot perform object/cell counting and use the resulting information to evaluate and visualize drug combination effects. Recently, the real-time moving object detector R-MOD has also been reported to offer label-free cell counting [17]. However, also this methodology has no obvious relation to drug combination analysis and relies on imaging flow cytometry, where suspension rather than adherent cell cultures are used. Moreover, the images analyzed are non-complex, as they contain a relatively small number of freely floating cells against a homogeneous background.

### Higher-order drug combinations

The use of higher-order drug combination regimens for complex diseases is following an increasingly upward trend [1, 2, 8]. For instance, cocktails of several drugs used in the context of metronomic chemotherapy have recently shown promising clinical results [6]. Moreover, polytherapies in the form of higher-order combinations, such as the anti-cancer protocols CUSP9 [3, 4, 18] for recurrent glioblastoma (GBM) and MEMMAT [5] for recurrent medulloblastoma, have already entered the clinic. Last but not least, there are continuous and joint efforts, such as the ReDO project [7], which are seeking for novel and affordable multidrug treatments by repurposing well-known and well-characterized drugs.

At the same time, there are still very few extensive reports from systematic large-scale in vitro studies of higher-order drug combinations [1, 2, 8]. The vast majority of multidrug regimens are the result of mainly in vivo studies, without first being subject to any kind of preceding detailed in vitro evaluation. In general, exhaustive in vitro experiments that assess all plausible subsets of the employed single drugs are required in order to disentangle higher- from lower-order effects [1, 8]. Apart from disease related insights, such an exhaustive approach would indicate which drugs seem to be most clinically relevant; patients should not be treated with multiple drugs when the desirable effects emerge merely from smaller subsets of them in combination [2].

The few aforementioned efforts for higher-order drug combination analysis have resulted in end point quantitative frameworks that can also disentangle higher- from lower-order drug effects [1, 2, 8]. However, they employ mathematical models, such as Bliss [19] and Loewe [20], which rely on specific assumptions and have their roots in toxicology. Although well-established, this type of toxicology-rooted synergy analysis may be completely misleading in a pharmacological context, where the goal is to identify drug combinations that exhibit large therapeutic windows [21]. Moreover, synergy analyses in general is non-trivial to formulate and employ with time series data, including the TLVM measurements used here. In such cases, multivariate data analysis methods seem more straightforward to employ in order to identify characteristic response behaviors as well as their associated drugs and/or drug combinations. As a first step towards this unexplored direction, we propose here such an approach that performs temporal data mining and is able to disentangle higher- from lower- and single-drug effects, without requiring any specific assumption about the drug interactions.

#### Exhaustive drug combination experiments

An exhaustive drug combination experiment is defined here to cover all possible different subsets of combinations among a panel of pre-selected drugs at one fixed concentration each. Given *N*_*d*_ pre-defined drugs, the number of experimental wells required for performing an exhaustive experiment up to order *c* can be expressed as:

1 $$ N_{w}(N_{d}, c) = \sum_{i = 1}^{c}{\binom{N_{d}}{i}}   $$

**Fig. 1:**
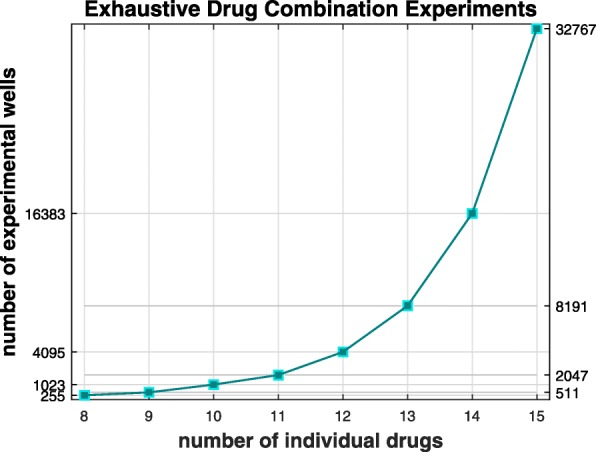
Experimental Capacity for Exhaustive Layouts. The experimental capacity required for performing exhaustive drug combination experiments grows rapidly with respect to the number of the individual drugs used. However, the graph shows that it is feasible to perform exhaustive experiments in one 384-well plate for up to 8 drugs

Thus, if *N*_*d*_=8 drugs are selected to modulate 8 different targets related to the disease of interest, a single exhaustive experiment exploring all plausible ways of perturbing these targets requires *N*_*w*_(8,8)=255 wells. Such an exhaustive experiment offers maximum resolution of the combinatorial space and requires advanced data analytics. Although such brute force experiments may become expensive, only one 384-well plate is needed for up to *N*_*d*_=8 drugs (Fig. 1). Notably, the use of multiple concentrations per drug requires much larger experimental capacity, but such a set up does not align with an exhaustive drug combination experiment as defined above and thus, it is not satisfied by eq. (1).

There is no reported methodology, so far, for automated design and label-free quantitative microscopy based processing of exhaustive drug combination experiments. Setting up such a methodological tool, which offers reproducible and traceable experiments by performing quality control (QC) at several levels and requiring very few human interventions, is highly needed. It could facilitate and accelerate large-scale higher-order drug combination experiments as well as generate useful data for iterative [22, 23] and in silico methods [13, 14].

### COMBImage2

Motivated by this background, we developed COMBImage2 (Table 1); a parallel computational framework for higher-order drug combination analysis that includes automated plate design, matched filter based object counting and temporal data mining. It consists of 6 different modules in total, which are briefly presented below:

1. COMBO-Pick automatically generates 384-well randomized layouts for any type of drug combination experiments by requiring only a simple user-defined text specification file.
2. COMBO-V offers cell viability and synergy (end point) analyses and visualization. The current version of COMBO-V is able to analyze higher-order and exhaustive drug combination experiments by extending our previously reported scaled Bliss and therapeutic synergy analyses [9].
3. COMBO-C offers automated quantification and visualization of temporal changes in cell growth/confluence. Apart from an improved foreground segmentation approach and the ability to analyze higher-order and exhaustive drug combination experiments, it has also been equipped with inter-plate QC procedures used when several replicate plates are employed.
4. COMBO-M offers automated quantification and visualization of temporal changes in cell morphology. The current updated version of COMBO-M provides alternative visualization as temporal curves and it is capable of analyzing higher-order and exhaustive drug combination experiments as well.
5. COMBO-MF offers automated detection, counting and visualization of objects present in the TLVM movies that look like apoptotic cells, using a linear 2-dimensional matched filter approach.
6. COMBO-Mine offers data fusion and temporal data mining for all different extracted response patterns. In this way, it is able to identify prototypical response behaviors over time in order to disentangle higher- from lower- and single-drug effects in a data driven way.

The tailor made image processing algorithms of COMBImage2 are implemented using the MapReduce programming model [24] with the goal to offer fast and scalable analyses independently of instruments, infrastructures and applications [9]. COMBImage2 is distributed as a package of 6 standalone applications for Windows together with all raw data of the corresponding case study [25–27].

### Case study

To demonstrate the potential of COMBImage2, we designed a semi-exhaustive drug combination experiment, using the CUSPv4 protocol [18], currently used in the clinic for recurrent GBM. More precisely, we studied for the first time, all 246 combinations of order 4 or lower in addition to the 9 single drugs. The effects were evaluated on a drug sensitive clonal culture of glioma-initiating cells (GICs) established from GBM patient tumor samples [28]. Our results suggested that there were only two main categories of behavioral patterns primarily induced by single drugs. In particular, Disulfiram (Dis) seemed to be the main player of one category, since it was part of all other drug combinations regardless of order. The corresponding phenotypic effects included increased changes in cell morphology and increased numbers of apoptotic-like cells early on, as well as almost zero cell survival. Similarly, we identified higher-order drug combinations, such as the 4-order combination consisting of Minocycline (Min), Dis, Sertraline (Ser) and Quetiapine (Que), which seemed to slightly boost the effect of Dis alone. In the second main category, all the corresponding multi- and single-drug responses had very similar behavior to untreated cells.

### Organization of the paper

The rest of this paper is organized as follows. **Results:** Methodological and pharmacological results related to the case study are presented; **Discussion:** The general methodological and pharmacological findings are discussed and summarized together with corresponding limitations; **Conclusions:** The importance and novelty of this work are clearly stated; **Materials and Methods:** Details related to the performed wet lab experiments, improved QC procedures, higher-order synergy analysis, tailor made image processing algorithms and temporal data mining are provided.

## Results

### Assay quality control

#### Intra-plate QC

COMBImage2 performs intra-plate QC in order to robustify the analysis within an experimental plate. The intra-plate QC procedure is fully automated and incorporated in all different computational modules. The corresponding algorithm is identical to the one reported in our previous work [9]. Briefly, it checks if at early (ideally untreated) time points, all experimental wells have similar feature vectors (i.e., hierarchical histograms). After an automated comparison, the wells that have deviating feature vectors are excluded from all subsequent analyses as they contain artifacts/noise that may falsify the results and corresponding interpretations. Notably, the cut-off threshold for the similarity is determined automatically, as described in our earlier study [9]. For this task, we ideally suggest the recording of one untreated time frame. However, if this is not possible, we at least require a very early treated time point, so that it is reasonable to assume that there are not yet any visible treatment effects. For instance, in this case study (Additional file 1: Figure S1), the first treated time frame (4*h* after drug addition) was used for the intra-plate QC, due to limited experimental capacity that did not allow earlier image recording.

#### Inter-plate QC

Inter-plate image QC is a novel feature of COMBImage2 and more specifically of COMBO-C, which calculates and visualizes changes in cell growth over time. This novel feature is developed and incorporated in order to robustify the analysis among replicate plates, before any further joint analysis. Only experimental wells that have successfully passed the preceding intra-plate QC (Additional file 1: Figure S1) are qualified for the subsequent inter-plate QC. The main idea behind the latter one is that replicate measurements with high variability should not be merged (Additional file 1: Figure S2). Notably, the cut-off threshold regarding the inter-plate variability is automatically determined by means of resampling (see “Methods” section, Additional file 1: Figure S3).

### COMBO-Pick for automated design of experiments

**Fig. 2:**
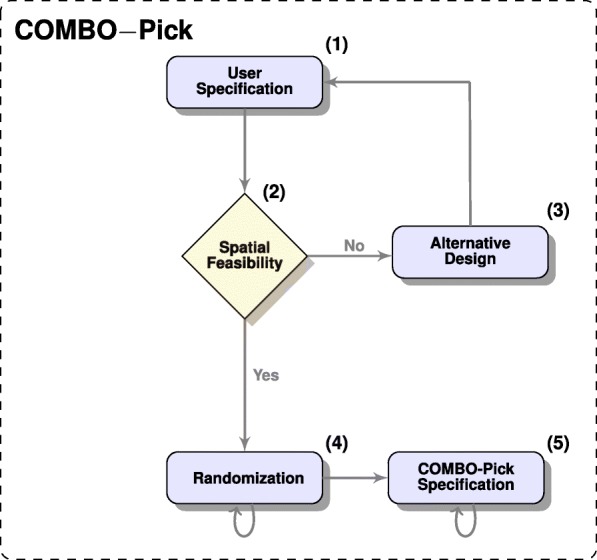
COMBO-Pick flowchart. **(1)** A user-defined text specification file is imported; **(2)** Spatial feasibility control for 384-well format allowing at least 40 untreated wells is performed; **(3)** Alternative spatially feasible designs are suggested to the user; **(4)** Randomization of well destinations; **(5)** A plate destination specification for either exhaustive or pairwise drug combination experiments, compatible with Bridge, is produced per plate. **4**-**5** are repeated independently for all replicate plates, as specified by the user in (**1**)

**Fig. 3:**
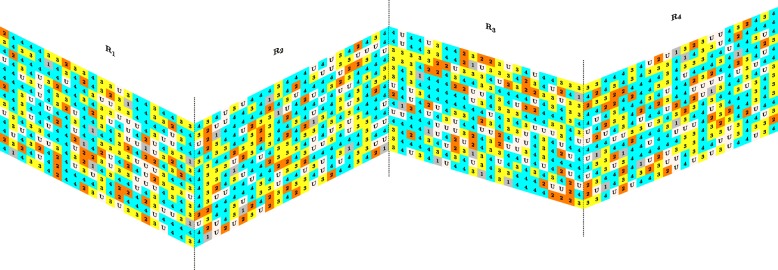
Randomized Plate Designs by COMBO-Pick. The pilot study was replicated 4 times using a differently randomized layout each time; *R*_1_,*R*_2_,*R*_3_,*R*_4_. Each layout consists of 5 different groups of wells based on the number of combined drugs: gray: 1; orange: 2; yellow: 3; cyan: 4; white: no drugs/untreated

COMBO-Pick is an experimental module (Fig. 2) that offers automated 384-well plate design (Additional file 1: Figure S4) and can be used with programmable acoustic liquid handling technologies. Currently, it is compatible with an in-house application, Bridge [29], which generates the corresponding transfer schemes for acoustic liquid dispension in an Echo 550 (Labcyte Inc., Sunnyvale, CA). COMBO-Pick makes efficient use of the plate by accommodating as many drug combination experiments as possible, while also including a large number of untreated control wells needed for reliable statistical analyses. Furthermore, the design is randomized, meaning that each experiment (i.e, drug/drug combination/untreated cells) has a randomly selected position in the plate, which is different across replicate plates (Fig. 3). The randomization procedure aims at eliminating potential spatial effects that may propagate during replication. In other words, if the experimental noise is spatially dependent, then the different replicates of the same experiment will be subject to (nearly) independent noise terms that can be filtered (often via averaging) in order to reduce experimental variability.

COMBO-Pick requires a single specification text file from the user, where the experiment is described in a particular way (Additional file 1: Figure S5). COMBO-Pick checks the spatial feasibility of this specification under the condition that at least 40 untreated wells must be accommodated per plate, in addition to the specified drugs/drug combinations. When the aforementioned criterion is not fulfilled, COMBO-Pick suggests alternative solutions by expanding the design in more than one plates. A spatially feasible user specification (Additional file 1:Figure S5) produces randomized plate layouts (Fig. 3), which are finally exported as destination plate specifications for Bridge [29] providing information about compound names, destination wells and final concentrations.

### COMBO-V for higher-order combinations

COMBO-V (Additional file 1: Figure S15) is a module for cell viability and synergy (end point) analyses. As reported in our recent work [9], it offers both target and reference cell focused synergy analyses, according to the Bliss model and the recently reintroduced therapeutic window concept [21]. Moreover, these two synergy scores were further refined by us [9], in order to account for ambiguities that arise when the same value is obtained for very different drug combination effects. Finally, a resampling based statistical analysis is employed for the synergy scores, so as to determine how likely these values may appear by random chance [30]. Here, we generalize our previously reported methodology for evaluating higher-order drug combinations (see “Methods” section) and performing inter-plate analyses. Since the current case study did not include a reference toxicity model, only results from the Bliss synergy analyses are provided (Additional file 1: Figure S6-S7), although no outstanding synergies were found (Additional file 1: Table ST1). In terms of the particular case study, the absence of synergy is apparent already by looking at the corresponding cell viability analysis (Additional file 1: Figure S16). There, Dis alone resulted in very low survival index (≈10*%*), while all drug combinations that were associated with values at the same low level contained Dis, suggesting absence of synergy. However, here we employed Bliss synergy analysis, in order to show how COMBO-V can be used for higher-order combination experiments.

### COMBO-C for higher-order drug combinations

COMBO-C (Additional file 1: Figure S8) is a module for cell confluence/growth analyses. As reported in our recent work [9], it offers quantification and visualization of temporal changes in cell growth (Additional file 1: Figure S9). The MapReduce implementation [24] provides fast analyses and potential for scalability if the data volume becomes too big for the memory of a single computer. Here, we generalize this methodology for all kinds of higher-order drug combinations including exhaustive experiments. Furthermore, another important improvement of COMBO-C is the ability to perform inter-plate QC, as described in a previous section above, by employing (non-parametric) resampling statistics (see “Methods” section). Notably, this inter-plate QC procedure of COMBO-C is employed for the corresponding inter-plate analyses of all modules.

### COMBO-M for higher-order drug combinations

COMBO-M (Additional file 1: Figure S10) is a module for morphology based analyses of drug effects. As reported in our recent work [9], it offers quantification of temporal changes in cell morphology which is currently represented in the form of hierarchical histograms. The feature extraction is parallelized using the MapReduce programming model [24], which also enables a grid search based parameter optimization of the two parameters (i.e., scale reduction of resolution, number of bins) for the histograms. Here, we generalize this methodology for higher-order drug combinations including (semi-)exhaustive experiments and provide a new more convenient way of visualizing the results as temporal curves. Moreover, COMBO-M is now able to perform inter-plate analyses when several replicate plates are employed for the same experiment (Additional file 1: Figure S11).

### COMBO-MF

**Fig. 4:**
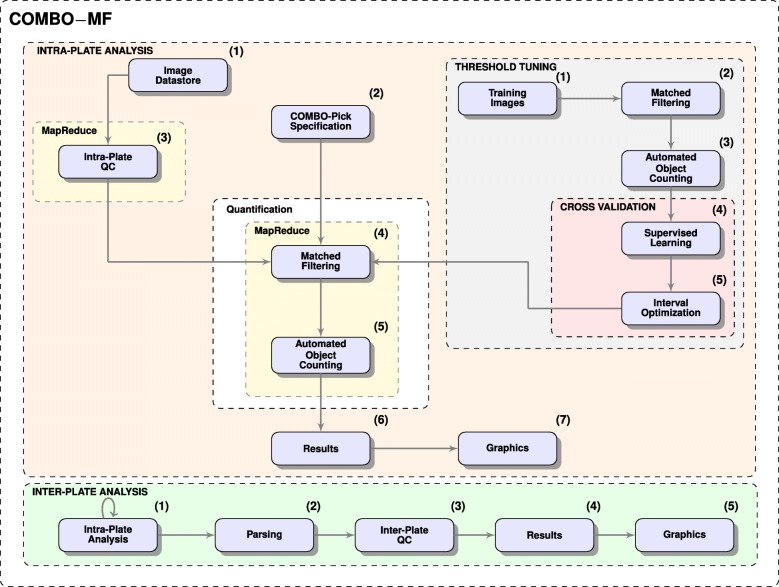
COMBO-MF Flowchart. **THRESHOLD TUNING:(1)**-**(3)** Matched filtering on training images; **(4)**-**(5)** Cross validation for optimal detection threshold. **INTRA-PLATE ANALYSIS:(1)** Image datastore selected by the user; **(2)** COMBO-Pick specification imported by the user; **(3)** MapReduce-based intra-plate quality control; **(4)**-**(5)** MapReduce-based quantification of apoptotic-like cells; **(6)** Table (CSV) with results; **(7)** Temporal graphics (EPS, PDF). **INTER-PLATE ANALYSIS:(1)** Intra-plate analysis employed separately for all replicates; **(2)** Results from **(1)** gathered and parsed; **(3)** Outlier removal based on the Inter-Plate QC as performed by COMBO-C; **(4)** Table (CSV) with merged inter-plate replicate values; **(5)** Temporal graphics (EPS, PDF)

**Fig. 5:**
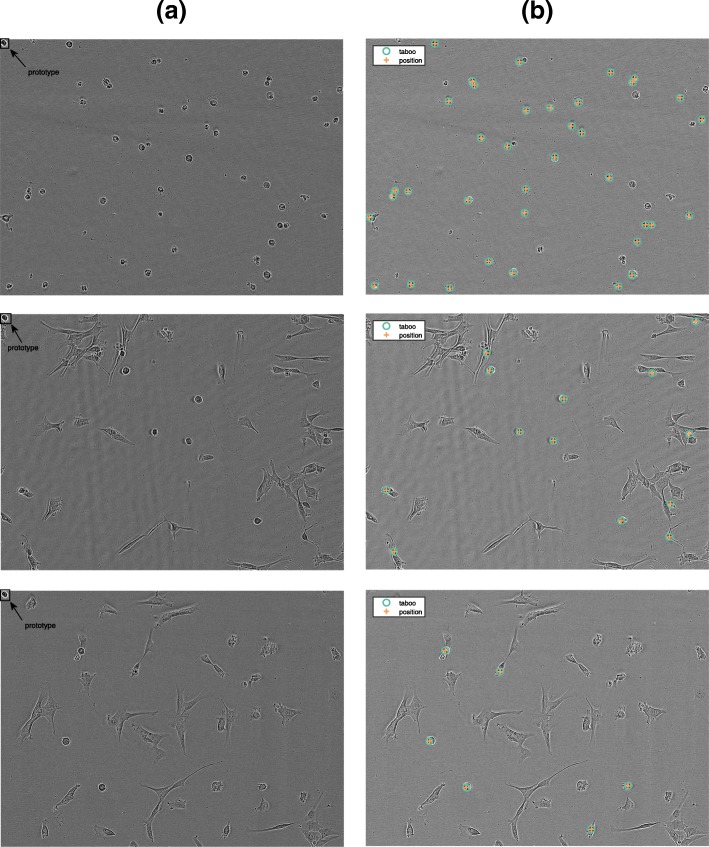
Apoptotic-like Object Counting. **(a)** Raw images where the prototypic object of size 33×32 pixels is overlaid on the left upper corner; **(b)** Prototypic-like detected objects. The green circles and orange crosses correspond to the detections made by the taboo- and position-based counting algorithms, respectively

COMBO-MF (Fig. 4) offers a MapReduce implementation of an optimized matched filter based image processing algorithm (see “Methods” section). Although it is currently adjusted to detect and count apoptotic-like cells present in phase-contrast images from large cell populations (Fig. 5) in 384-well format, its functionality can easily be extended for other objects of interest, given a corresponding prototype specification. It is able to evaluate drug combination experiments of any order, including pairwise and exhaustive plate layouts, as well as perform inter-plate analyses when several replicate plates are employed (Additional file 1: Figure S14).

#### Apoptotic-like object counting

**Fig. 6:**
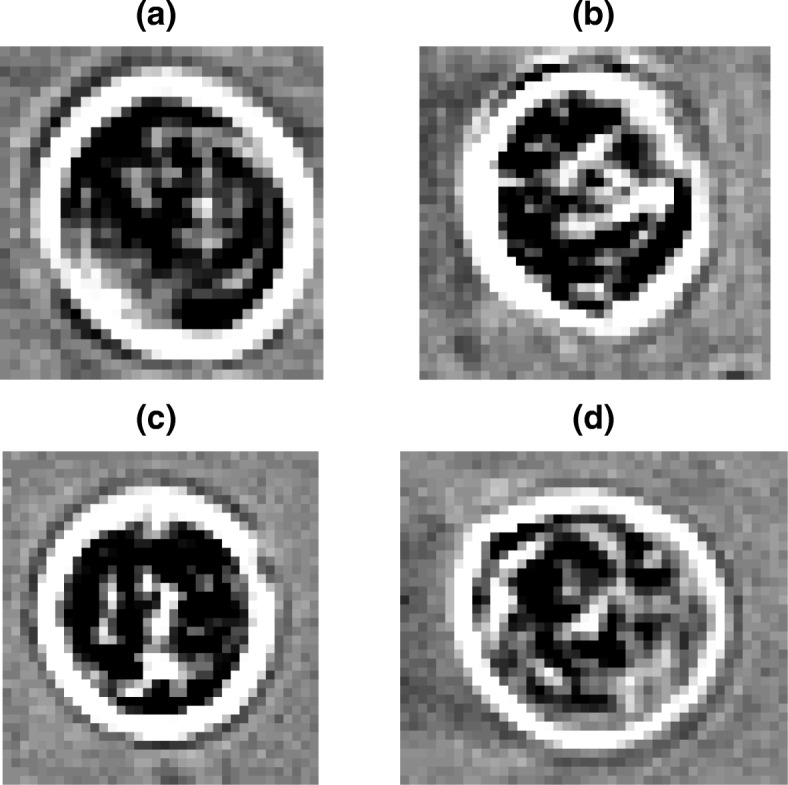
Prototypical Objects. COMBO-MF was evaluated using four similar prototypical objects of sizes: **(a)** 33×32 pixels; **(b)** 38×40 pixels; **(c)** 38×36 pixels and **(d)** 37×43 pixels

COMBO-MF builds on our earlier work [15], where the detections were made at the level of individual pixels, by now offering quantification at the level of distinct objects, meaning counting of apoptotic-like cells. This is performed by means of two new tailor made algorithms (Algorithms 1 and 2). In contrast to our previous work [15] where the prototypic object was manually designed, now it is selected by the user as a local image patch from the corresponding image library. In order to facilitate this selection, we suggest that the user should look at wells for which the cell viability is low and the change in cell morphology is high, after running COMBO-V and COMBO-M, respectively. In this way, there should be multiple images with such apoptotic-like formations to choose from. The size should be close to the average size in the population of apopototic-like cells observed. Here, we show that the choice of the prototypical object among several similar options has almost no impact on the results of COMBO-MF (Additional file 1: Figure S12), by employing four different prototypes (Fig. 6). Given that all four prototypical objects yield very similar results (Additional file 1: Figure S12), the first one (Fig. 6a) was further used for the main analysis.

The *MapReduce* programming model [24] is employed for the matched filter signal processing along with the aforementioned object counting procedure. In particular, the *Map* function employs the two different object counting methods per time frame (Algorithms 1 and 2), while the *Reduce* function produces the final average results per experimental well. By default, the current MapReduce implementation is executed on a local parallel pool by deploying all available cores of the machine used. Here, 8 cores were used (see “Methods” section). For the current study, the average running time per 384-well plate (5236 images) was approximately 5 min.


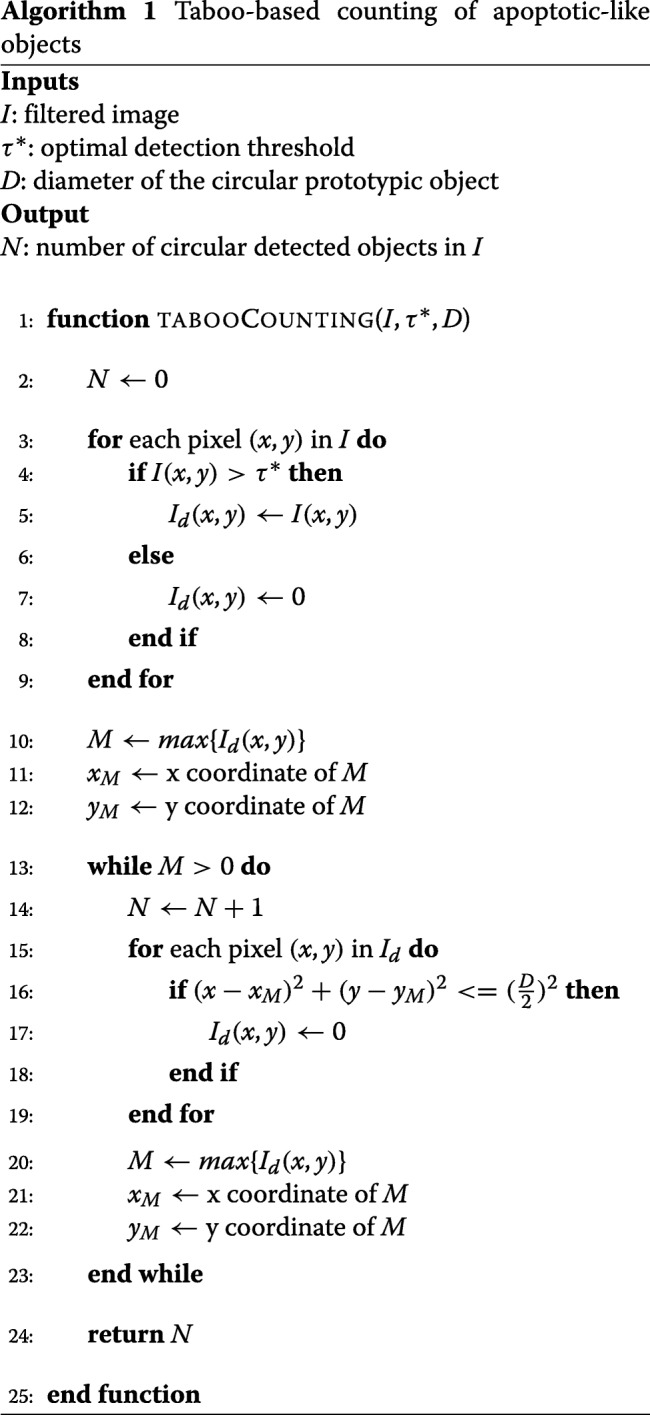



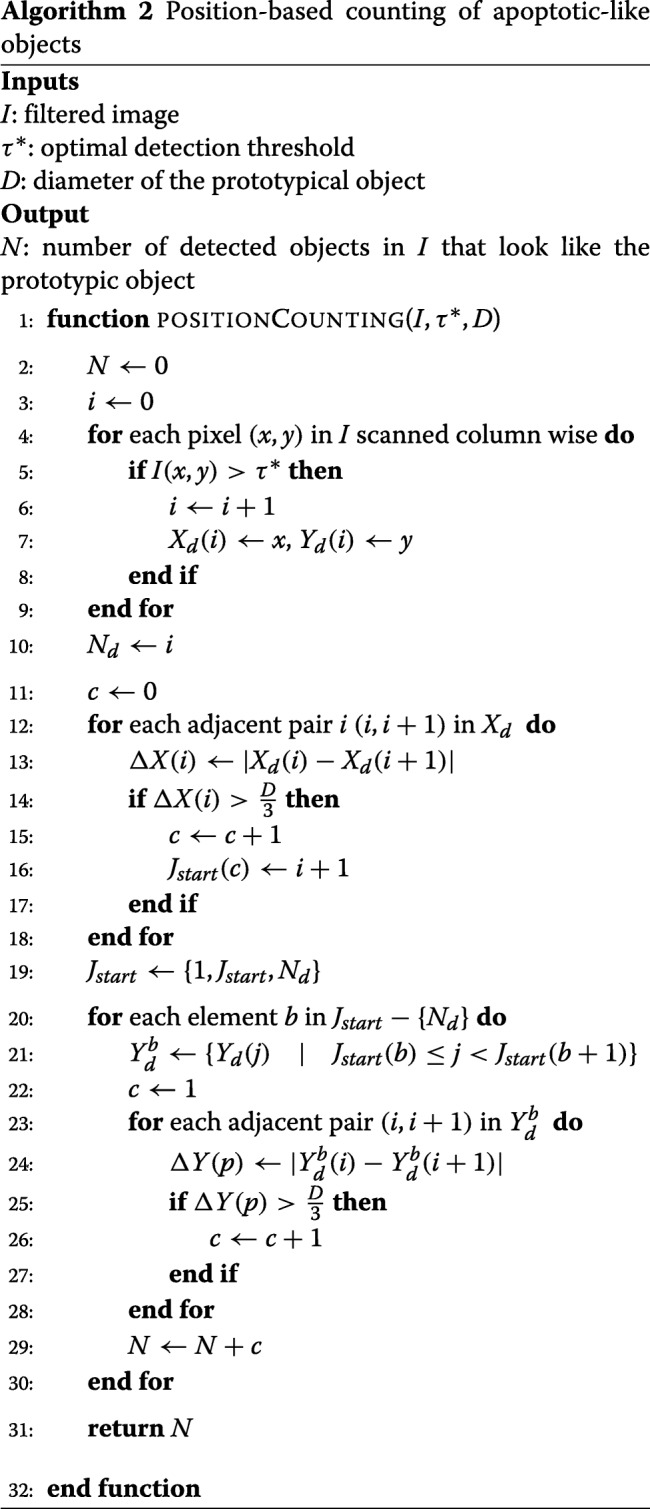


#### Matched filter threshold tuning

The optimal detection threshold for the matched filter is adaptively determined by supervised learning using an interval optimization search (see “Methods” section, Additional file 1: “Threshold Tuning Explained” section, Figure S13 and Algorithm SA1-SA2). To reduce the risk of overfitting, 4-fold cross validation was employed and repeated 2 times. The optimal detection threshold value is determined as the median value of the thresholds obtained from the cross validation partitions (Additional file 1: Figure S13). In terms of the current case study, 8 training images were used; 2 from each replicate plate [25]. This threshold tuning procedure is a new development, which serves the need to provide individual object/cell counts. In order to increase the chances of having a successful cross validation based threshold tuning procedure, we recommend the use of at least 8 training images, where each one of them contains simultaneously non-apoptotic- and apoptotic-like objects.

### COMBO-Mine

**Fig. 7:**
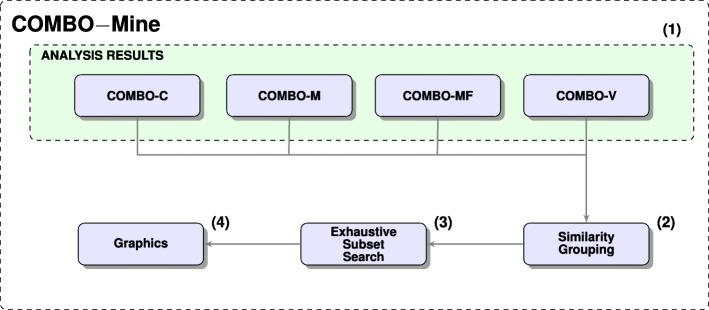
COMBO-Mine Flowchart. **(1)** Results from all four analysis modules, COMBO-C, COMBO-M, COMBO-MF and COMBO-V, are required; **(2)** The extracted response patterns from **(1)** are organized into groups with similar behavior. For this grouping, multilevel K-means clustering is currently employed; **(3)** The smallest non-redundant subset of drugs and/or drug combinations for each group is identified by an exhaustive algorithmic search as shown in Fig. 8. **(4)** Each group is visualized by the corresponding average temporal profiles as determined by **(2)** and represented by the smallest non-redundant subset obtained from **(3)**

COMBO-Mine (Fig. 7) is a tailor made computational methodology for temporal drug combination analysis, which performs data fusion and mining for the extracted response patterns; changes in cell confluence/growth (Additional file 1: Figure S9), changes in cell morphology (Additional file 1: Figure S11), apoptotic-like cell counts (Additional file 1: Figure S14) and cell viability (Additional file 1: Figure S16).

**Fig. 8:**
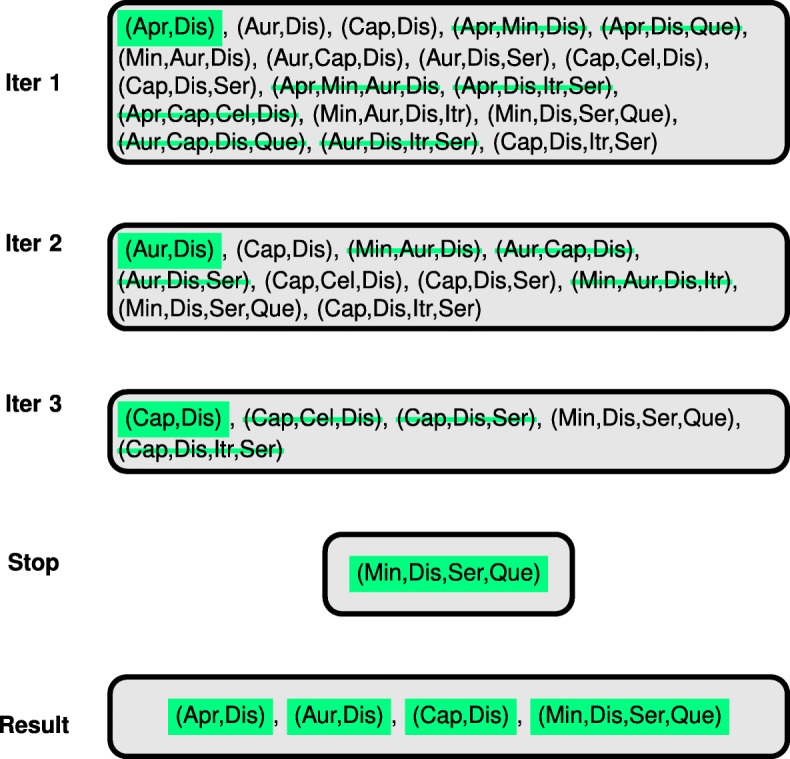
Exhaustive Subset Search. Each group of the extracted response patterns is only represented by the smallest set of drugs and/or drug combinations that uniquely explains all of them in the same group. To illustrate the employed algorithmic procedure of this search, an example of our case study is used. In each iteration (iter 1−3), the drug combination of the lowest order is traced in all remaining ones of higher order. All higher-order combinations that include the to-be-traced lower-order combination, are subsequently removed. Thus, when the algorithm terminates, the corresponding non-redundant set of drug and drug combination names is formed

#### Discovery and interpretation of prototypical response patterns

COMBO-Mine (Fig. 7) currently employs top down hierarchical clustering using K-means at each level (see “Methods” section, Additional file 1: Figure S17-S18) to discover prototypical response behaviors. The main idea is to organize the large combinatorial response space into groups with distinct prototypical behaviors that the user is able to characterize as either interesting or uninteresting without any particular model assumption. For each (sub-)group identified, an exhaustive subset search is performed to narrow down the unique single drugs and/or drug combinations that induce the corresponding prototypical temporal (and viability) profiles. Notably, all drugs/drug combinations that belong to such a unique subset are ranked equally much as they are part of the same group.

This exhaustive subset search helps to disentangle higher- from lower- and single-drug effects. To exemplify, let us assume that there are two drugs *X* and *Y* at concentrations *c*_*X*_ and *c*_*Y*_, respectively. If the response patterns *f*(*c*_*X*_) and *f*(*c*_*X*_,*c*_*Y*_) for *c*_*X*_ and the combination concentration (*c*_*X*_,*c*_*Y*_) form together a particular group/cluster *A* with an average (prototypical) response pattern $\widetilde {f}_{A}$, then the exhaustive search identifies *c*_*X*_ as representative of $\widetilde {f}_{A}$. Similarly, in the more concrete example related to the current case study (Fig. 8), only the drug names are illustrated, since each drug was used at one fixed concentration (see “Methods” section).

### Case study

COMBImage2 was employed in the context of a semi-exhaustive in vitro study of the higher-order CUSP9v4 cocktail [18]. In this study, we evaluated for the first time all possible combinations of up to order 4 on an in vitro clonal culture of GICs. One fixed concentration was used for each one of the 9 individual drugs (see “Methods” section), resulting in 246 different combinations; 36 of order 2, 84 of order 3 and 126 of order 4. The experiment was replicated 4 times so as to perform more meaningful and reliable statistical analyses. COMBO-Pick was employed in order to design and produce the plate layouts for this experiment (Fig. 3), which were used for the acoustic liquid drug transfer (see “Methods” section). Each and every of the four plates were first analyzed separately (intra-plate analysis) and then jointly (inter-plate analysis) by the computational modules COMBO-V (Additional file 1: Figure S15), COMBO-C (Additional file 1: Figure S8), COMBO-M (Additional file 1: Figure S10) and COMBO-MF (Fig. 4). At the end, COMBO-Mine (Fig. 7) was employed to combine all these results and perform temporal data mining in order to identify prototypical response behaviors and corresponding drugs and/or drug combinations.

**Fig. 9:**
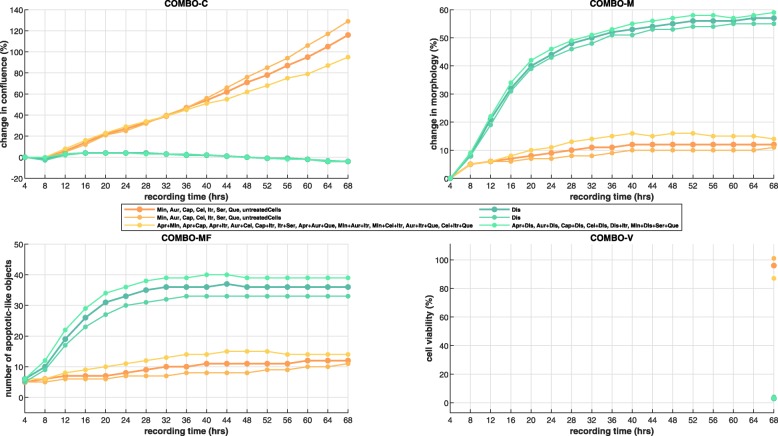
COMBO-Mine Results. The employment of COMBO-Mine in the context of the current CUSP9 case study revealed 2 main groups with 2 subgroups each. Each (sub)group is visualized by the corresponding four average response patterns (three image based temporal profiles and one endpoint cell viability value) and characterized by the smallest non-redundant set of drugs and/or drug combinations in it

**Fig. 10:**
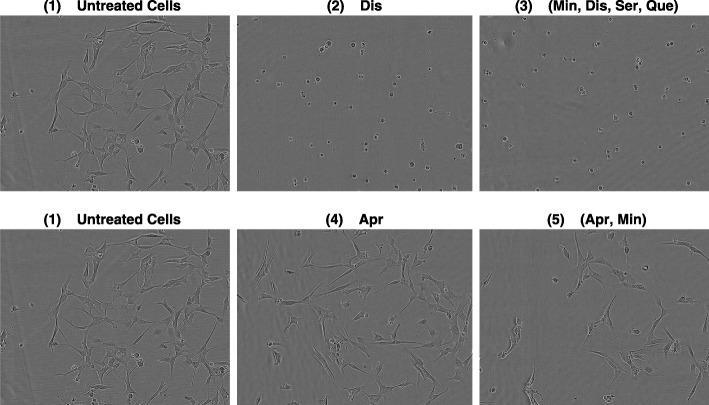
Selected Microscopy Images for the CUSP9v4 case study. Example images of the sensitive GIC clone at *t*=68*h* after drug addition, based on the similarity grouping of the extracted response patterns as provided by COMBO-Mine. The images show: **(1)** untreated cells (shown twice), and cells treated with **(2)** Dis ; **(3)** (Min, Dis, Der, Que) ; **(4)** Apr ; **(5)** (Apr, Min)

COMBO-Mine revealed two main response patterns/groups (Fig. 9). In particular, Dis was part of all drug combinations in one of the groups, regardless of order. This suggested that Dis alone was responsible for the corresponding prototypical response behaviors; total inhibition of cell growth, increased changes in cell morphology and increased number of apoptotic-like cell counts already at 12*h*, as well almost zero cell survival at 68*h* after drug addition (Figs. 9 and 10). Inside the “Dis” group, two subgroups were identified. One of them included drug combinations with slightly larger response behaviors, especially in terms of apoptotic-like cell counts (Fig. 9). The smallest unique (non-redundant) subset for this subgroup included 6 drug combinations; [Aprepitant (Apr), Dis], [Auranofin (Aur), Dis], [Captopril (Cap), Dis], [Celecoxib (Cel), Dis], [Dis, Itraconazole (Itr)] and (Min, Dis, Ser, Que). The second main group demonstrated uninteresting response patterns as they resembled those of untreated cells.

## Discussion

COMBImage2 is a parallel and modular computational framework for drug combination analysis of any order that includes automated plate design, matched filter based object counting and temporal data mining (Table 1). The drug combination effects are analyzed by means of label-free quantitative video microscopy jointly together with conventional end point measurements. COMBImage2 is able to extract multiple temporal cellular phenotypes, including changes in cell growth and morphology as well as apoptotic-like cell counts. In addition to higher-order Bliss synergy (end point) analyses, it provides a temporal data mining approach, which is able to organize the drug combination effects into groups with similar response behaviors. In this way, it offers a straightforward and data driven method for identifying characteristic response behaviors over time as well as their associated drugs and/or drug combination. This helps the user to disentangle higher- from lower- and single-drug effects by visually identifying interesting drug induced behavioral patterns without requiring any specific assumption about the drug interactions. Different aspects and limitations of COMBImage2 are discussed below.

### Pharmacological aspects

The potential of COMBImage2 was demonstrated in the context of a semi-exhaustive in vitro experiment using the CUSP9v4 cocktail [18]. More precisely, the effects of all possible combinations of order 4 or lower were studied using a drug sensitive GIC clone [28]. The drug concentrations (Table 2) were determined by means of a separate dose response experiment. The goal from this pre-analysis was to fix the drug concentrations at clinically relevant levels [31–34], while achieving very little individual drug effects in vitro (i.e., no lower than 90% cell viability compared to untreated controls). The fixed concentration of 0.67*μM* (Table 2) for Dis resulted in approximately 95% cell viability in the initial dose response experiment, while the same concentration resulted in approximately 10% cell viability in the main exhaustive experiment (Additional File 1: Figure S16). This unexpectedly strong cytotoxic effect of Dis made the pharmacological results less interesting than expected and desired. Despite these complications, COMBImage2 was successfully employed to design and analyze a semi-exhaustive drug combination experiment, showing great potential for similar applications in general. As a final note, we did not use the standard-of-care drug, Temozolomide (TMZ), as in the corresponding clinical set up, since our goal was to disentangle any combination effects of the 9 repurposed drugs.

**Table 2:** Drugs and concentrations. Fixed concentrations for the individual drugs of the CUSPv4 protocol [18] used for the case study

Drug	Concentration (*μ*M)	Abbreviation
Aprepitant	2.6	Apr
Minocycline	0.44	Min
Auranofin	0.15	Aur
Captopril	0.12	Cap
Celecoxib	1.6	Cel
Disulfiram	0.67	Dis
Itraconazole	0.3	Itr
Sertraline	0.5	Ser
Quetiapine	3	Que

### Computational aspects

#### Automated design of drug combination experiments

COMBO-Pick (Fig. 2) is a new module, which automatically generates experimental layouts for 384-well plates based on a simple user-defined text specification file. It is able to produce plate layouts for any kind of drug combination experiments that can be used by programmable acoustic liquid handling technologies. Although COMBO-Pick is currently made compatible with an in-house program, Bridge [29], it can be easily adjusted to other similar softwares. The automated plate design offers multiple advantages, mainly including efficiency, flexibility, scalability and traceability. By offering optimized and randomized plate layouts, the experimental capacity is efficiently used, while potential spatial effects are reduced. Notably, the pairwise layouts reported in our earlier study [9], which were manually designed, have been optimized by COMBO-Pick, meaning that there is now larger spatial capacity per 384-well experimental plate than before.

#### Inter-plate image QC

Although replicating an experiment across several different plates might be expensive, it is very useful for increasing the reliability of the subsequent data analytics and statistics. More precisely, such independent inter-plate measurements allow us to take into account the observed experimental variability, in order to avoid potential misinterpretations. For this task, COMBImage2 employs (non-parametric) resampling statistics, which are easy to implement given the current computer power, while at the same time are model independent.

#### Apoptotic-like object counting

COMBO-MF (Fig. 4) offers automated label-free quantification and visualization of apoptotic-like cell counts for video microscopy based drug combination analysis. The cell counting goes far beyond the previously mentioned LFAD [15], where the detections were made only at the level of individual pixels. In order to provide consistent automated and observed cell counts, the detection threshold for the matched filter is tuned by cross validation based supervised learning, using only 8 training images that have been manually annotated [25]. We show that the exact choice of the prototype is not crucial for the performance (Fig. 6, Additional File 1: Figure S12). It should be noted that this approach is not expected to provide near perfect counting, but rather a sufficiently good way of localizing drug treated wells with high number of apoptotic-like cells along with associated temporal information. The MapReduce implementation of COMBO-MF offers scalability when the data volume is too big to fit into the memory of a single computer. It would be very interesting to compare the matched filter based cell counting of COMBO-MF with convolutional neural network approaches reported, for example the already mentioned R-MOD [17]. However, in this case there is a requirement for at least 200 manually annotated training images along with 5 million parameters to be tuned, which do not allow a direct quantitative comparison in terms of this work.

#### Discovery and characterization of prototypical reponse patterns

COMBO-Mine (Fig. 5) performs data fusion and mining of all extracted response patterns; changes in cell growth and morphology, apoptotic-like cell counts and cell viability. In this way, it offers a data driven way of disentangling higher- from lower-order and single-drug effects, when their evaluation includes time series in addition to end point measurements.

It currently employs top down hierarchical clustering using K-means at each level (see “Methods” section) to discover prototypical response behaviors. Although other machine learning methods should also be evaluated in the future to explore potential improvements, here we introduce, for the first time, a tailor made computational methodology for temporal drug combination analysis based on quantitative video microscopy. The main idea is to organize the large combinatorial response space into distinct groups that the user is able to characterize as either interesting or uninteresting without any particular model assumption about the drug interactions. In order to demonstrate the potential of this temporal data mining approach against conventional synergy (end point) analyses, we compare below the corresponding results from our CUSP9v4 case study.

As shown by our Bliss synergy analyses (Additional file 1: Figure S6-S7), six drug combinations were identified as weakly synergistic (Additional file 1: Table ST1), since the corresponding scaled Bliss index values (see “Methods” section) were very close to zero (Additional file 1: Figure S6-S7 and Table ST1). COMBO-Mine was able to detect the absence of synergy by partitioning the combinatorial space into two main groups. In particular, one of them showed very similar response patterns to untreated cells (Fig. 9). Not unexpectedly, this group included all six drug combinations identified as weakly synergistic by the aforementioned Bliss analysis (Additional file 1: Table ST1). The second group exhibited single-drug effects, including inhibition of cell growth, large morphological changes, induction of apoptosis and very low cell viability, all seemingly induced by Dis alone. Thus, being able to discover and characterize prototypical response patterns seems much more informative than ranking the drug combinations with a particular model based synergy score. In conclusion, the employment of COMBO-Mine in the context of similar drug combination studies may reveal interesting and unique single-/lower-/higher-order drug effects, which might be missed by conventional synergy end point analyses.

## Conclusions

In brief, we report:

- COMBImage2; a modular, parallel, robust, automated and instrument independent computational framework for drug combination analysis of any order that includes automated plate design, matched filter based object counting and temporal data mining. In particular, compared to COMBImage [9], COMBImage2 offers:
  - randomized and optimized design for drug combination experiments of any order and type, including pairwise and exhaustive layouts, in 384-well format
  - refined higher-order Bliss synergy analyses coupled with (non-parametric) resampling statistics
  - robust inter-plate analyses by employing resampling based quality control
  - quantification and visualization of temporal changes in cell growth and morphology as well as apoptotic-like cell counts
  - tailor made computational methodology for temporal drug combination analysis that helps the user to discover prototypical response behaviors and disentangle higher- from lower- and single-drug effects
- A small pilot in vitro study, which did not provide any outstanding pharmacological findings, as only single-drug effects were observed for the semi-exhaustive experiment of the CUSP9v4 protocol [18]. However, it clearly shows how COMBImage2 can be generally used to design, robustly analyze and visualize higher-order drug combination experiments, based on label-free quantitative video microscopy and single end point (cell viability) measurements.

COMBImage2 is the first methodological tool reported so far, which is able to automatically design and process higher-order exhaustive drug combination experiments using joint label-free video microscopy and conventional end point measurements. It uniquely combines refined higher-order drug combination analyses, quantitative video microscopy, laboratory automation, quality control procedures, resampling statistics, MapReduce parallelization and temporal data mining. All these non-trivial components have been for the first time integrated into a generic and modular framework that is able to provide reliable, robust, information rich and scalable drug combination analyses of any order. Apart from the extraction of multiple temporal cellular phenotypes, which goes far beyond the currently available methods, COMBImage2 also provides a novel data mining approach for evaluating the response patterns and disentangling higher- from lower- and single-drug effects without requiring any specific assumption about the drug interactions. Furthermore, such a versatile video microscopy oriented framework is likely to enable systematic large-scale drug combination studies not only related to cancer, which is the main practice today, but also other diseases. For example, it could be employed for antimicrobial susceptibility testing to identify promising higher-order combinations of antibiotics and disease models where (de)differentiation of the cells is of interest. Taken together, COMBImage2 demonstrates a novel methodological framework with the potential to improve, guide and accelerate early stages of drug combination discovery and development.

## Methods

In the following, details regarding the wet lab experiments, image-based assay QC procedures and all computational methods developed and employed are presented in detail.

### Wet lab

#### Cell cultures

The GBM clonal cell culture, *U*3065−*c*271 [28], was cultured in neural stem cell media (1:1 mix of DMEM-F12 GlutaMAX medium and Neurobasal medium (Life Technologies/GIBCO-Invitrogen) containing 1% penicillin G/streptomycin sulfate (Sigma-Aldrich, St. Louis, MO), supplemented with B-27 without vitamin A (1:50; Invitrogen), N2 supplement (1:100; Invitrogen), 10 ng/mL EGF and 10 ng/mL FGF-2 (PeproTech, Rocky Hill, NJ). Cells were seeded in poly-L-ornithine (P4957, Sigma-Aldrich) and laminin (L2020, Sigma-Aldrich) coated 384-well plates (164688, Thermo Fisher Scientific) at a density of 1000 cells/well using a BioMek 4000 (Beckman Coulter). All cells were seeded 24 h prior to treatment with compounds.

#### Chemical compounds

The CUSP9v4 protocol [18] was employed in the context of the current case study. For each one of the 9 single drugs, a fixed concentration was determined by an initial in-house dose response experiment and reported blood-plasma levels [31–34]. The goal was to choose for each drug a concentration that induces very little effect in vitro (i.e., approximately 90% cell viability), while at the same time lying within in vivo levels. Table 2 includes the fixed concentrations for all 9 drugs.

#### Label-free video microscopy recording

Phase-contrast time-lapse microscopy images were acquired using the IncuCyte FLR (Essen BioScience Inc.) located inside the incubator. The microscope had a 20× objective with the ability to capture high quality phase-contrast microscopy images, 1040×1392 pixels each. Seventeen frames/images per experimental well were acquired, one every 4*h*. The total size of image data per 384-well plate was 5.6GB (5236 images).

#### Experimental capacity

For the case study, 255 drug treated wells were required per 384-well plate, according to eq. (1), in addition to 53 untreated control wells (Additional file 1: Figure S4). The experiment was performed in quadruplicate (Fig. 3).

#### Assay for determination of survival index

Cell survival was determined by means of the Fluorometric Cytotoxicity Assay (FMCA), [35, 36]. Cell survival for a particular combination concentration vector **c**_*n*_ of *n* drugs, known otherwise as survival index and denoted here as *S*, is calculated as:

2 $$ S(\mathbf{c}_{n}) = \frac{f(\mathbf{c}_{n}) - \tilde{f}_{blank}}{\tilde{f}_{control} - \tilde{f}_{blank}}  $$

Here *f*(**c**_*n*_) corresponds to the fluorescence signal from the experimental well of **c**_*n*_, while $\tilde {f}_{blank}$ and $\tilde {f}_{control}$ denote the median fluorescence signal from the blank and growth control wells, respectively. For drugs causing growth inhibition and/or cell killing the range of *S* spans from 0 to 1 indicating minimal and maximal cell survival respectively, compared to untreated cells.

#### Bliss synergy analysis

The conventional Bliss synergy analysis as well as our recently reported rescaling method [9] were generalized the equations for higher-order drug combinations. For a particular combination concentration vector **c**_*n*_ of *n* drugs the conventional Bliss index *B* is defined as:

3 $$ B(\mathbf{c}_{n}) = \prod_{i = 1}^{n}S(c_{i}) - S(\mathbf{c}_{n})  $$

Here, $\prod _{i=1}^{n}S(c_{i})$ and *S*(**c**_*n*_) denote the expected (according to Bliss) and observed survival index values, respectively. *B*(**c**_*n*_)=0 means independent action of the *n* drugs, whereas *B*(**c**_*n*_)>0 and *B*(**c**_*n*_)<0 are defined as synergy and antagonism, respectively. The values of *B* range from [−1,1] indicating maximal Bliss antagonism and synergy, respectively. However, *B* can be ambiguous; the same *B* value can be achieved by several different pairs of $\prod _{i = 1}^{n}S(c_{i})$ and *S*(**c**_*n*_) values. To discriminate between such cases, avoid misinterpretations and maintain many of the original properties of *B*, we defined a novel scaled version [9], denoted *B*_*S*_, as:

4 $$\begin{array}{*{20}l} B_{S}(\mathbf{c}_{n}) =& B(\mathbf{c}_{n})\cdot \left[1 - min\left\{\prod_{i = 1}^{n}S(c_{i}), S(\mathbf{c}_{n})\right\}\right]\\ =& \left\{ \begin{array}{ll}  B(\mathbf{c}_{n})\cdot \left[1 - S(\mathbf{c}_{n})\right] & \quad \text{if}\ B(\mathbf{c}_{n}) > 0\\ 0 & \quad \text{if}\ B(\mathbf{c}_{n}) = 0\\ B(\mathbf{c}_{n})\cdot \left[1 - \prod_{i = 1}^{n}S(c_{i})\right] & \quad \text{if}\ B(\mathbf{c}_{n}) < 0 \end{array}\right.  \end{array} $$

Here, $min\Big \{\prod _{i=1}^{n}S(c_{i}), S(\mathbf {c}_{n})\Big \}$ denotes the minimum value among the expected and observed survival index values, respectively. Thus, in the case of synergy, *B*_*S*_ suppresses cases associated with high observed survival values, while in the case of antagonism it suppresses cases associated with high predicted survival values [9]. Notably, similar rescaling methods have been independently proposed in the field of genetics [37], but no comparison with our approach has been done so far.

### Assay QC

#### Inter-plate variability estimate

**Fig. 11:**
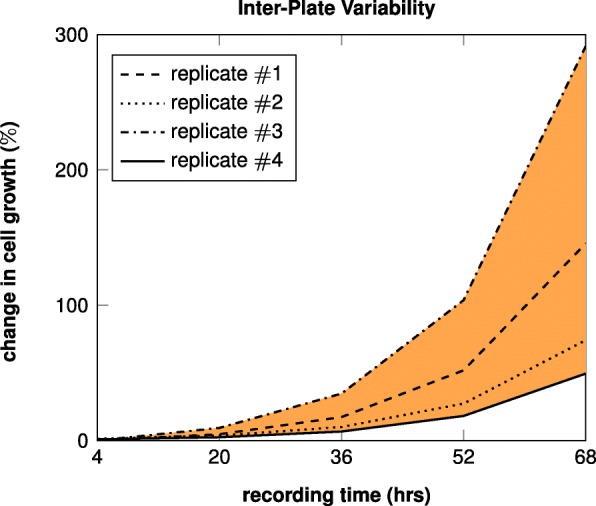
Inter-Plate Variability Estimate. The area spanned by the 4 growth curves is calculated and used as the inter-plate variability estimate for a particular well *w*. For convenience, this example illustrates the simplest case, where one replicate has the biggest change in cell growth for all time points

Using the relative change as defined in (8), the change in cell growth over time for a particular well *w* can be obtained by the corresponding growth curve (Fig. 11). When *w* is replicated in more than one plates, the inter-plate variability estimate denoted as *V*_*w*_, is here calculated as the area between the replicate growth curves:

5 $$ V_{w} = \sum_{t}\Big(\Delta C_{w}^{max}(t) - \Delta C_{w}^{min}(t)\Big)  $$

Here $\Delta C_{w}^{max}$ and $\Delta C_{w}^{min}$ denote the maximum and minimum changes among replicates for time point *t*. Using a simple example case with 4 inter-plate replicates (Fig. 11), the area *V*_*w*_ shown in orange is calculated as:

6 $$ V_{w} = \sum_{t \in T}\Big(\Delta C_{w}^{(3)}(t) - \Delta C_{w}^{(4)}(t)\Big), \hspace{3mm} T = \{4,8, \cdots, 68\}  $$

Notably, Fig. 11 corresponds to the simplest case when one replicate has the largest change in cell growth for all time points. In most cases, the maximum value corresponds to different replicates for different time points.

##### Null hypothesis significance testing for inter-plate variability

In order to estimate the statistical distribution for the inter-plate variability, *N*=10000 simulations were performed using resampling. In particular, for each simulation *j*, one untreated well was randomly selected with replacement from each of the 4 plates. Then, the associated variability *V*_*u*,*j*_ was calculated using (5). This resulted in a null distribution:

7 $$ \hat{V}_{u} = \{V_{u,1}, \cdots, V_{u,N}\}   $$

which was used to define a QC threshold for the inter-plate variability (Additional file 1: Figure S3). More specifically, the 95^*th*^ percentile *τ*_95_ was employed here as the null threshold, meaning that for higher values, the null hypothesis was rejected. Such a rejection indicates an outlier, since the observed inter-plate variability is atypical and seldom occurring by random chance (Additional File 1: Figure S2). As a consequence, the probability of false alarm (i.e., the probability of falsely detecting a non-outlier as an outlier) was 5%. Notably, this probability as well as the number of simulations *N* for the null hypothesis significance testing are user-defined parameters in the general framework.

### Novel image processing and analysis features

#### Adaptive foreground segmentation

COMBImage uses threshold-based segmentation in order to divide the images into foreground and background pixels [9]. This binary classification offers noise reduction, since all background pixels are ignored from subsequent computational analyses. Here, we report an advancement of our previous work by following the same notation. Instead of a user-defined background intensity interval *τ*, COMBImage2 currently provides an adaptive multi-thresholding method for determining the extreme points of *τ*. In particular, the Otsu’s method [38] is applied to the background intensity distribution twice, independently for the two regions defined by splitting the pixels according to the global background intensity estimate *μ*_*b*_.

#### Quantification of changes in cell growth and morphology

As introduced in COMBImage [9], the change in cell growth/confluence *c*_*w*_(*t*_*i*_) for a particular experimental well *w* and time point *t*_*i*_ relative to the first time point *t*_0_, is defined as:

8 $$ \Delta C_{w}(t_{i}) = \frac{c_{w}(t_{i}) - c_{w}(t_{0})}{c_{w}(t_{0})}   $$

Similarly, for a particular experimental well *w* and time point *t*_*i*_, morphological features in the form of hierarchical pixel histograms (PHHC) are extracted as a column vector **h**_*w*_(*t*_*i*_). In order to quantify and express relative changes in morphology between consecutive time points, we introduce a new measure denoted *ΔM*_*w*_ defined as:

9 $$ \Delta M_{w}(t_{i}) = \frac{{\mathbf{h}_{w}(t_{i}) - \mathbf{h}_{w}(t_{0})}_{1}}{{\mathbf{h}_{w}(t_{0})}_{1}}   $$

*ΔM*_*w*_ gives the value zero at *t*_0_ and subsequently positive values at later time points if the changes in morphology are increasing. This relative measure establishes a reference value *ΔM*_*w*_(*t*_0_)=0 and compensates for differences in cell seeding that often make the results between different experimental wells incomparable.

#### Matched filter for object counting

##### The linear matched filter

A linear two-dimensional matched filter is used to detect apoptotic-like cells. The detector corresponds to a sliding image patch of size *N*×*N* and therefore *N*^2^ filter coefficients. In the form of a *N*^2^×1 dimensional column vector **r**, the matched filter calculates the optimal test statistic for discrimination between the two hypotheses:

10 $$\left\{ \begin{array}{ll} H_{0}: & \quad \mathbf{r} = \mathbf{b} + \mathbf{n}\\ H_{1}: & \quad \mathbf{r} = \mathbf{s} + \mathbf{n} \end{array}\right.   $$

Here, **b** denotes the background, **s** denotes the signal prototype to be detected and $\mathbf {n} \sim \mathcal {N}(0, \, \mathbf {C})$. Given a filter coefficient vector **w** and a particular local image patch **r**, the output of the filter is:

11 $$ y = \mathbf{w}^{\intercal} \mathbf{r}   $$

where y is a scalar product corresponding to the value of the central pixel in **r**. The filter coefficient vector **w** is calculated as:

12 $$ \mathbf{w} = \mathbf{C}^{-1}(\mathbf{s} - \mathbf{b}) = \mathbf{s} - \mathbf{b}  $$

Since the goal here was to detect isolated apoptotic-like cells in a non-structured background, the covariance matrix was set equal to the identity matrix, **C**=**I**. Details about the derivation of (12) are provided in Additional file 1: “Matched Filter” section.

##### Object counting

For a given threshold of the matched filter output, object counting is performed using two in-house developed algorithms referred to here as taboo- and position-based counting, respectively (Fig. 5 and Algorithms 1 and 2). Briefly summarized, the basic idea of the taboo method is to start with the pixel centered at the largest matched filter output, increase the counter by one, and then put the pixel as well as its closest neighbors on a taboo list, until all pixels in the image are covered. For the position-based counting algorithm, the basic idea is to first find pixel intervals along the horizontal dimension of the image that correspond to detected objects. Then the same procedure is employed along the vertical dimension but only for each of the horizontal intervals already identified as containing at least one object. Finally the total number of object containing intervals along the horizontal direction are summed together, in order to determine the total number of objects detected.

##### Threshold tuning

The optimal detection threshold for the matched filter in (12) is determined adaptively by supervised learning using a customized interval optimization search (Additional file 1: Algorithm SA1-SA2). This allows for automated and generic tuning of the threshold, regardless of application. For the supervised learning, the actual number of prototypic-like objects present in the training images are specified by visual inspection, while the predicted number is automatically retrieved by the two aforementioned tailor made algorithms (Algorithms 1 and 2). The average difference between the observed and predicted number of objects is used as the loss function, which is initially minimized among a set of starting points and then within a data dependent search interval.

To reduce the risk of overfitting during the tuning of the matched filter threshold, cross validation is employed (Additional file 1: Figure S13). For each partition *j*, an optimal detection threshold $\tau _{j}^{*}$ is found and the corresponding test error $\widetilde {f}_{test}(\tau _{j}^{*})$ is calculated using the images belonging to the leave-out fold. At the end, a decision is made accordingly; if $\underset {j}{\text {median}}\left \{\widetilde {f}_{test}\left (\tau _{j}^{*}\right)\right \}$ is greater than 5, the framework terminates due to overfitting and suggests re-training using more data. Otherwise, the final optimum is obtained as $\underset {j}{\text {median}}\{\tau _{j}^{*}\}$, which is used for further processing. Notably, the number of corresponding partitions for the cross validation is a user-defined parameter in the general framework.

#### Mining of extracted response patterns

In order to organize and thereby simplify the temporal drug combination effects in terms of the all four different phenotypic responses, multilevel K-means clustering is used. This algorithm is employed as implemented in MATLAB R2018b [39] with the default similarity measure, which is the sum of squared errors (SSE). More precisely, this clustering method is known as K-means++ [40], which combines an improved initialization method with “Lloyd’s generalized algorithm” [41]. All extracted *n*-dimensional profiles per well are normalized with their own standard deviation before being stacked in a common *N*×1 vector, where *N*=4*n* and 4 reflects the four different analysis results; changes in cell growth (COMBO-C), changes in morphology (COMBO-M), apoptotic-like cell counts (COMBO-MF) and cell viability (COMBO-V). Notably, each cell viability value is firstly transformed into a *n*×1 vector with the same value in all elements, in order to match the length of the corresponding image-based time series data.

The number of clusters *K* to be used in each hierarchical level is determined among a set of different values and in particular, *K*_*s*_={1,2,⋯,10}. The clustering procedure is repeated *R* (user-defined) times for all different values of *K* in the set *K*_*s*_ (Additional file 1: Figures S17–S18). For each repetition *r* of a particular *K*, the corresponding SSE denoted as $\hat {E}$ is calculated as:

13 $$ \hat{E}_{K, r} = \sum_{k = 1}^{K}\sum_{i = 1}^{N_{k}} {\mathbf{x}_{k,i}^{(r)} - \mathbf{c}_{k}^{(r)}}^{2}, \hspace{2mm} K \in K_{s}  $$

Here, $\mathbf {x}_{k,i}^{(r)}$ denotes the *N*-dimensional profile for repetition *r* of drug combination *i* that belongs to cluster *k* and $\mathbf {c}_{k}^{(r)}$ represents the *N*-dimensional centroid of cluster *k*. In order to express the decrease in SSE when transitioning between two consecutive *K* values, we define the corresponding relative change as:

14 $$ \Delta \hat{E}_{K-1\rightarrow K} = \frac{\hat{E}_{K} - \hat{E}_{K-1}}{\hat{E}_{K-1}}\cdot 100, \text{where}\ K = 2, \cdots, 10  $$

Here, $\hat {E}_{K}$ and $\hat {E}_{K-1}$ denote the two minima $\underset {r}{\text {min}}\{\hat {E}_{K, r}\}$ and $\underset {r}{\text {min}}\{\hat {E}_{K-1, r}\}$, respectively. The smallest *K* that results in SSE drop bigger than 20% compared to the previous value *K*−1, is selected (Additional file 1: Figure S17-S18). For a selected *K*, the partition corresponding to $\underset {r}{\text {min}}\{\hat {E}_{K, r}\}$ is further used.

### Programming environment and computing resources

COMBImage2 was developed in MATLAB R2018b [42]. The computations in the context of the current case study were performed on resources provided by the Swedish National Infrastructure for Computing (SNIC) at SNIC Science Cloud (SSC).

## Additional file

Additional file 1 Supplementary information. Additional text, results, figures and tables. (PDF 1519 kb)
