# Supplementary material for: COMBImage2: a parallel computational framework for higher-order drug combination analysis that includes automated plate design, matched filter based object counting and temporal data mining
Source: BMC Bioinformatics. 2019 Jun 4;20:304. doi: 10.1186/s12859-019-2908-0 (PMC6549340; doi:10.1186/s12859-019-2908-0)
Supplement: Supplementary file 1 — Supplementary information. Additional text, results, figures and tables. (PDF 1519 kb) [file 12859_2019_2908_MOESM1_ESM.pdf]

Supplementary Information

COMBImage2: a parallel computational framework for higher-order drug combination analysis that includes automated plate design, matched filter based object counting and temporal data mining

Efthymia Chantzi*[1], Malin Jarvius[1,2], Mia Niklasson[3], Anna Segerman[1,3], and Mats G. Gustafsson[1]

[1]Department of Medical Sciences, Cancer Pharmacology and Computational Medicine, Uppsala University, Uppsala, Sweden
[2]SciLifeLab Drug Discovery and Development, *In Vitro* and Systems Pharmacology Facility, Uppsala University, Uppsala, Sweden
[3]Department of Immunology, Genetics and Pathology, Rudbeck Laboratory, Uppsala University, Uppsala, Sweden

Contents

1 Matched Filter **1**
1.1 Decision Statistics . . . 1
1.2 Threshold Tuning Explained . . . 2
1.2.1 Starting Points . . . 2
1.2.2 Loss function . . . 3
1.2.3 Interval Optimization . . . 3

2 Results **4**

Appendices **20**

A Algorithms **20**

List of Figures

1 Intra-Plate QC . . . 4
2 Intra-Plate Analyses by COMBO-C . . . 5
3 Inter-Plate Quality Control by COMBO-C . . . 6
4 384-well plate format employed by COMBO-Pick . . . 6
5 User specification for COMBO-Pick . . . 7
6 Bliss Analysis . . . 7
7 Resampling Statistics for Bliss Synergy Analysis . . . 8
8 COMBO-C Flowchart. . . . 9
9 Inter-Plate Analysis by COMBO-C . . . 10
10 COMBO-M Flowchart. . . . 11
11 Inter-Plate Analysis by COMBO-M . . . 12
12 Four prototypical objects for COMBO-MF . . . 13
13 Cross Validation based Threshold Tuning . . . 14
14 Inter-Plate Analysis by COMBO-MF . . . 15

*corresponding author: efthymia.chantzi@medsci.uu.se

15 COMBO-V Flowchart 16
16 Inter-Plate Analysis by COMBO-V 17
17 K-means clustering 18
18 K-means sub-clustering 19

List of Tables

1 Bliss Synergy Scores 8

List of Algorithms

SA1 Tuning of the matched filter threshold 20
SA2 Find left and right extreme points for Algorithm SA1 21

1 Matched Filter

A linear two-dimensional matched filter is used to detect apoptotic-like cells. The detector corresponds to a sliding image patch of size $N \times N$ and therefore N^2 filter coefficients. In the form of a $N^2 \times 1$ dimensional column vector $\mathbf{r}$, the matched filter calculates the optimal test statistic for discrimination between the two hypotheses:

$$\begin{cases} H_0: & \mathbf{r} = \mathbf{b} + \mathbf{n} \\ H_1: & \mathbf{r} = \mathbf{s} + \mathbf{n} \end{cases} \tag{1}$$

Here, $\mathbf{b}$ denotes the background, $\mathbf{s}$ denotes the signal prototype to be detected and $\mathbf{n} \sim \mathcal{N}(0, \mathbf{C})$. Given a filter coefficient vector $\mathbf{w}$ and a particular local image patch $\mathbf{r}$, the output of the filter is:

$$y = \mathbf{w}^\intercal \mathbf{r} \tag{2}$$

1.1 Decision Statistics

H_1 is determined over H_0, if:

$$P(H_1|\mathbf{r}) > P(H_0|\mathbf{r}) \tag{3}$$

Using Bayes' theorem, the aforementioned probabilities can be derived as:

$$P(H_i|\mathbf{r}) = \frac{p(\mathbf{r}|H_i) \cdot P(H_i)}{p(\mathbf{r})}, \qquad i = \{0, 1\} \tag{4}$$

Thus, inequality (3) becomes:

$$\begin{aligned} \frac{p(\mathbf{r}|H_1) \cdot P(H_1)}{p(\mathbf{r})} &> \frac{p(\mathbf{r}|H_0) \cdot P(H_0)}{p(\mathbf{r})} \quad \Rightarrow \\ p(\mathbf{r}|H_1) \cdot P(H_1) &> p(\mathbf{r}|H_0) \cdot P(H_0) \quad \Rightarrow \\ \frac{p(\mathbf{r}|H_1)}{p(\mathbf{r}|H_0)} &> \frac{P(H_0)}{P(H_1)} \end{aligned} \tag{5}$$

The probability density function for the left part of inequality (5), under the assumption that $\mathbf{r} \sim \mathcal{N}(\mathbf{m}, \mathbf{C})$, is:

$$p(\mathbf{r}|H_i) = \frac{e^{-\frac{1}{2}(\mathbf{r}-\mathbf{m}_i)^\intercal \mathbf{C}^{-1}(\mathbf{r}-\mathbf{m}_i)}}{(2\pi)^{\frac{k}{2}} |\mathbf{C}|^{\frac{1}{2}}} \tag{6}$$

Here, $\mathbf{m}_0 = \mathbf{b}$ and $\mathbf{m}_1 = \mathbf{s}$ under H_0 and H_1, respectively. By plugging (6) in (5), we get:

$$\frac{e^{-\frac{1}{2}(\mathbf{r}-\mathbf{s})^\mathsf{T}\mathbf{C}^{-1}(\mathbf{r}-\mathbf{s})}}{e^{-\frac{1}{2}(\mathbf{r}-\mathbf{b})^\mathsf{T}\mathbf{C}^{-1}(\mathbf{r}-\mathbf{b})}} > \frac{P(H_0)}{P(H_1)}$$

$$\log\left(e^{-\frac{1}{2}(\mathbf{r}-\mathbf{s})^\mathsf{T}\mathbf{C}^{-1}(\mathbf{r}-\mathbf{s})+\frac{1}{2}(\mathbf{r}-\mathbf{b})^\mathsf{T}\mathbf{C}^{-1}(\mathbf{r}-\mathbf{b})}\right) > \log\left(\frac{P(H_0)}{P(H_1)}\right)$$

$$-(\mathbf{r}-\mathbf{s})^\mathsf{T}\mathbf{C}^{-1}(\mathbf{r}-\mathbf{s}) + (\mathbf{r}-\mathbf{b})^\mathsf{T}\mathbf{C}^{-1}(\mathbf{r}-\mathbf{b}) > 2\log\left(\frac{P(H_0)}{P(H_1)}\right)$$

$$\begin{aligned}-\mathbf{r}^\mathsf{T}\mathbf{C}^{-1}\mathbf{r} + \mathbf{r}^\mathsf{T}\mathbf{C}^{-1}\mathbf{s} + \mathbf{s}^\mathsf{T}\mathbf{C}^{-1}\mathbf{r} - \mathbf{s}^\mathsf{T}\mathbf{C}^{-1}\mathbf{s} + \mathbf{r}^\mathsf{T}\mathbf{C}^{-1}\mathbf{r}&\\ -\mathbf{r}^\mathsf{T}\mathbf{C}^{-1}\mathbf{b} - \mathbf{b}^\mathsf{T}\mathbf{C}^{-1}\mathbf{r} + \mathbf{b}^\mathsf{T}\mathbf{C}^{-1}\mathbf{b} &> 2\log\left(\frac{P(H_0)}{P(H_1)}\right)\end{aligned}$$

$$2\mathbf{r}^\mathsf{T}\mathbf{C}^{-1}\mathbf{s} - 2\mathbf{r}^\mathsf{T}\mathbf{C}^{-1}\mathbf{b} - \mathbf{s}^\mathsf{T}\mathbf{C}^{-1}\mathbf{s} + \mathbf{b}^\mathsf{T}\mathbf{C}^{-1}\mathbf{b} > 2\log\left(\frac{P(H_0)}{P(H_1)}\right)$$

$$\mathbf{r}^\mathsf{T}\underbrace{\mathbf{C}^{-1}(\mathbf{s}-\mathbf{b})}_{\mathbf{w}} > \underbrace{\log\left(\frac{P(H_0)}{P(H_1)}\right) + \frac{1}{2}\mathbf{s}^\mathsf{T}\mathbf{C}^{-1}\mathbf{s} - \frac{1}{2}\mathbf{b}^\mathsf{T}\mathbf{C}^{-1}\mathbf{b}}_{\tau} \tag{7}$$

The right part of inequality (7) represents the threshold τ, which determines H_1 over H_0. The left part corresponds to the filter coefficient vector defined as:

$$\mathbf{w} = \mathbf{C}^{-1}(\mathbf{s}-\mathbf{b}) \tag{8}$$

1.2 Threshold Tuning Explained

1.2.1 Starting Points

For each training image i, the fraction x_i in percent of the total image area covered by the observed prototypic objects is estimated as:

$$x_i = \frac{N_i^{obs} \cdot \alpha_{obj}}{\alpha_i} \cdot 100 \tag{9}$$

Here, N_i^{obs} denotes the number of observed prototypic objects, α_{obj} denotes the area covered by such a prototypic object and α_i the total area of the image. Then, the initial detection threshold τ_i for image i is determined as:

$$\tau_i = p_{100-x_i} \tag{10}$$

where p_{100-x_i} is the percentile of the "after filtering" intensity distribution that x_i belongs to. In other words, the fraction x_i of all pixels being above τ_i (ideally) corresponds to the N_i^{obs} objects. After applying (9) and (10), to all n training images, a set of initial guesses is defined as:

$$S_\tau = \{\tau_1, \tau_2, \cdots\cdots, \tau_n\} \tag{11}$$

which is split into m user defined equidistant values. The distance between two such consecutive values used to define the step of the employed interval optimization search is defined as:

$$\tau_{step} = \frac{\tau_{max} - \tau_{min}}{m} \tag{12}$$

Here, τ_{step} is the step between two consecutive searches, while τ_{max} and τ_{min} denote the maximum and minimum values in S_τ, respectively. For the case study, $m = 100$ was used.

1.2.2 Loss function

A detection threshold τ is evaluated on a set of n images by comparing the number of observed and predicted objects. The corresponding loss function $\widetilde{f}$ is defined as:

$$\widetilde{f}(\tau) = \frac{\|\mathbf{N}^{obs}(\tau) - \mathbf{N}^{pred}(\tau)\|_1}{n} \tag{13}$$

Here, $\mathbf{N}^{obs}$ and $\mathbf{N}^{pred}$ are n-dimensional column vectors, denoting the number of observed/actual and predicted/detected objects, respectively.

1.2.3 Interval Optimization

The set of initial guesses S_τ is exhaustively searched by minimizing $\widetilde{f}$ inside the shortest interval I bounding the initial minimum τ^*_{init}, thus defined as:

$$I = [\tau_L, \tau_R] \quad \text{where} \quad \{\tau_L, \tau_R \in S_\tau : \quad \widetilde{f}(\tau_L) > \widetilde{f}(\tau^*_{init}), \quad \widetilde{f}(\tau_R) > \widetilde{f}(\tau^*_{init})\} \tag{14}$$

where τ_L and τ_R are the closest left and right thresholds respectively, around τ^*_{init} among S_τ, where $\widetilde{f}$ is increasing. For a particular threshold value τ_k in I, the next evaluation at τ_{k+1} is calculated as:

$$\tau_{k+1} = \tau_k + \tau_{step} \quad \text{where} \quad \{\tau_k, \tau_{k+1} \in I\} \tag{15}$$

The optimal threshold τ^*_k is obtained by minimizing $\widetilde{f}$ in I, which is then further used for detecting and counting the prototypic-like objects in all filtered images. Thus, the corresponding optimization problem can be expressed as:

$$\tau^* = \min_{\{\tau_k \in I\}} \widetilde{f}(\tau_k) \tag{16}$$

2 Results

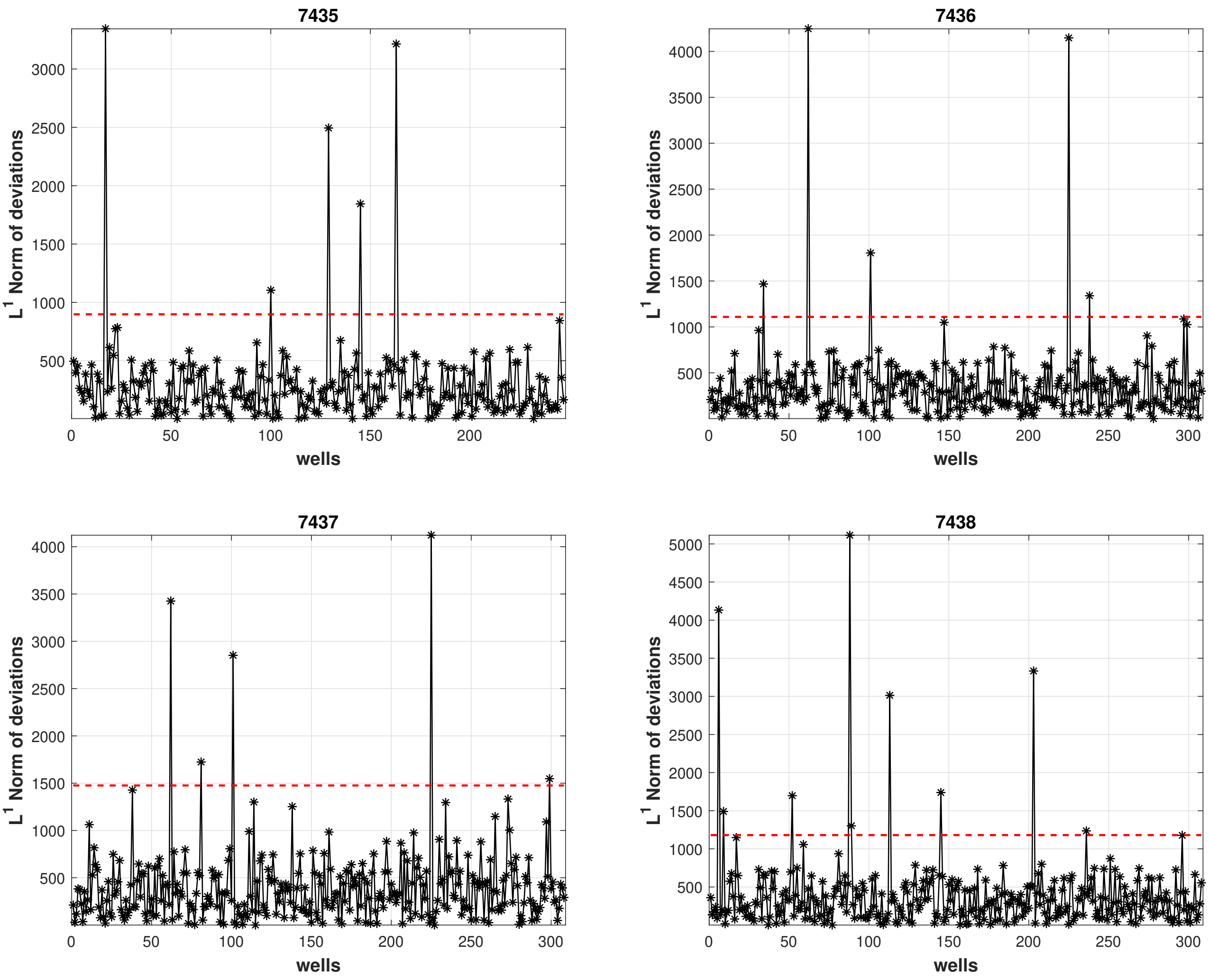

Figure S1: Intra-Plate QC for the four individual replicate plates (7435,7436,7437,7438) of the semi-exhaustive CUSP9v4 case study. All experimental wells lying above the red dotted line (automatically chosen cut-off threshold) were detected as outliers and were subsequently excluded from all subsequent analyses.

Barcodes: 7435 7436 7437 7438 (Date: 20170703)

Change in Confluence (%) : U3065-c271

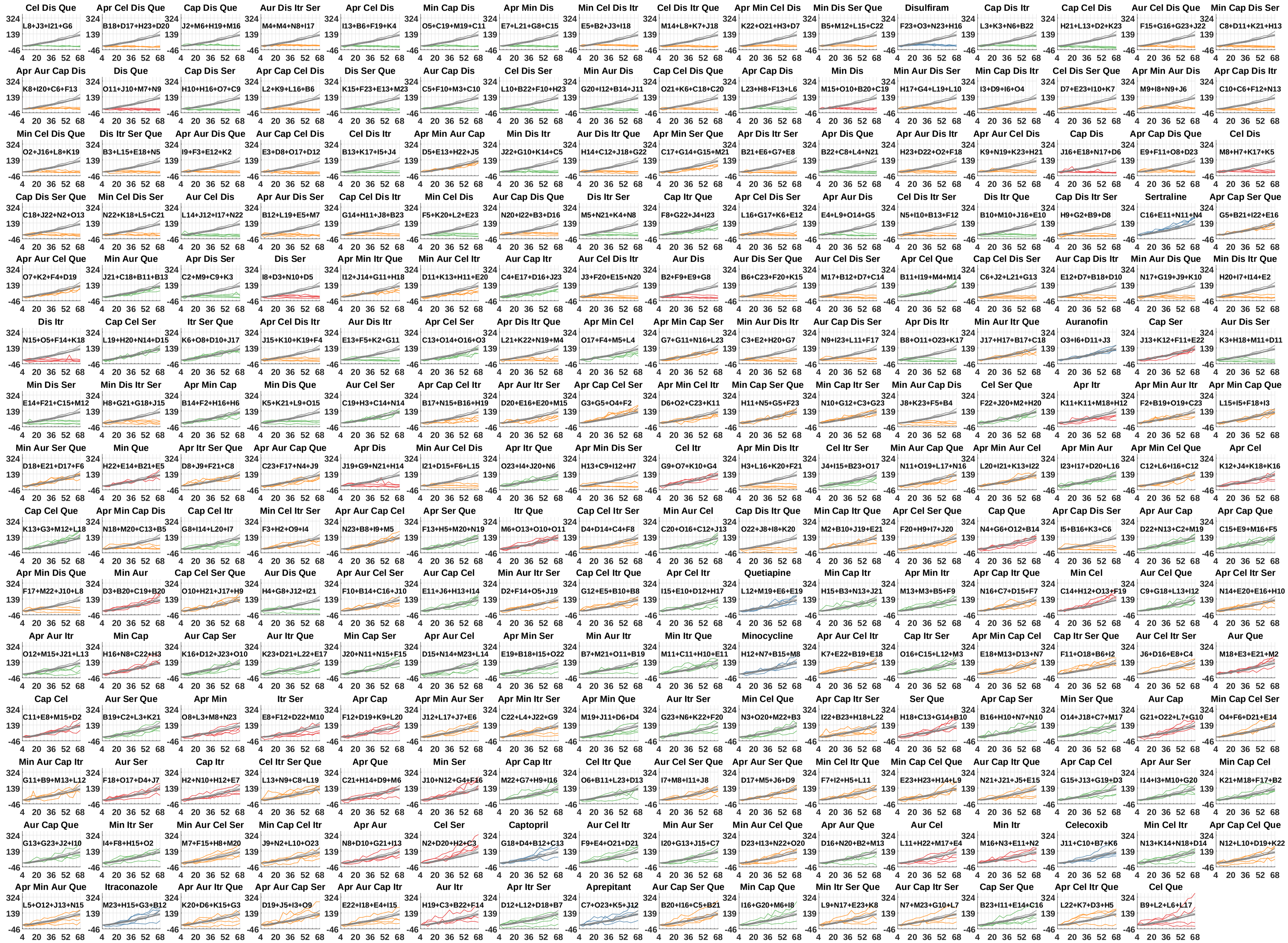

Figure S2: Growth curves for the sensitive GIC clone ($U3065 - c271$) in ascending order of inter-plate variability. The results of the 4 replicate plates for a particular treatment are jointly shown together with the corresponding median results for untreated cells (gray curves). The x-axis corresponds to the recorded time in hours, starting at $4h$ and ending at $68h$ after drug addition, respectively. The y-axis shows the cell growth (%) with respect to $4h$. The corresponding well names are shown in each subplot.

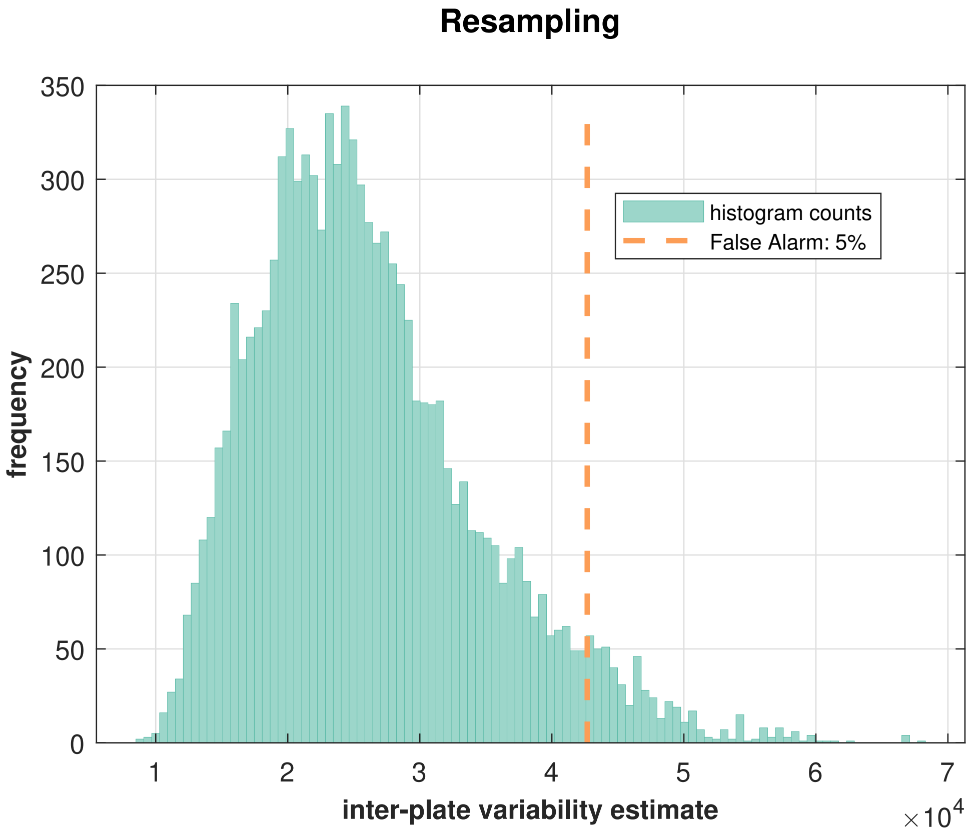

Figure S3: Histogram of estimated inter-plate variabilities used in the context of resampling based null hypothesis significance testing with 10000 simulations and 5% probability of false alarm. All inter-plate measurements with variability larger that this threshold were detected as outliers and they were excluded from all subsequent inter-plate analyses.

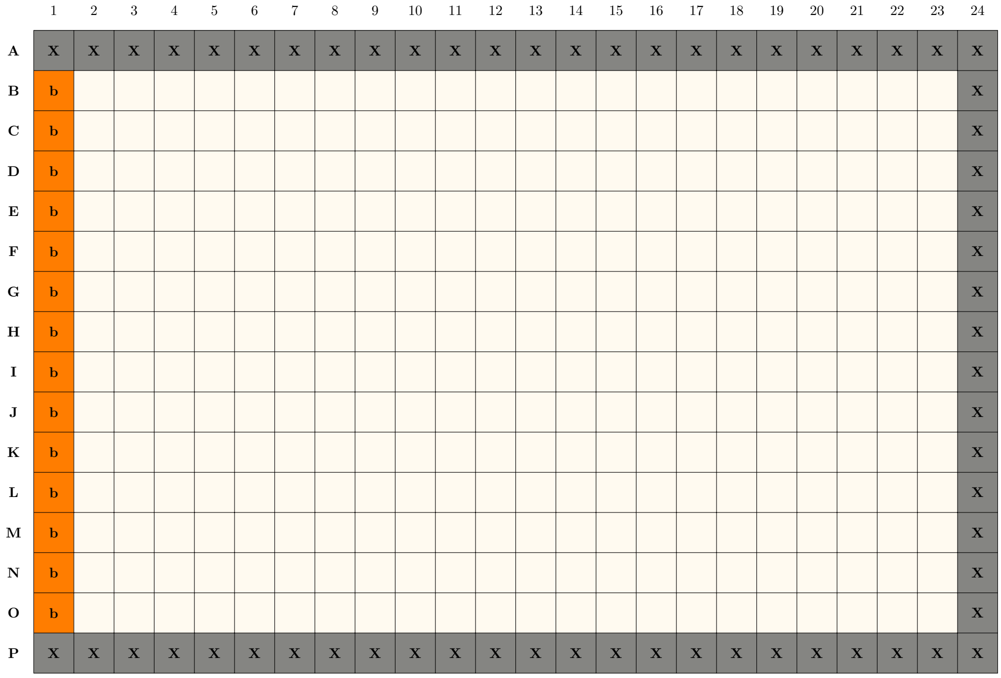

Figure S4: COMBO-Pick generates randomized experimental layouts in 384-well format with the following conventions: (i) B1-O1 are used as blank wells ; (ii) A1-A24, B24-O24 and P1-P24 are not used as destination wells in order to avoid potential drying issues. Thus, only the inner beige part, which consists in total of 308 wells is used by COMBO-Pick for the generation of randomized layouts.

```
x,1,Aprepitant,2.6,micromolar
x,1,Minocycline,0.44,micromolar
x,1,Auranofin,0.15,micromolar
x,1,Captopril,0.12,micromolar
x,1,Celecoxib,1.6,micromolar
x,1,Disulfiram,0.67,micromolar
x,1,Itraconazole,0.3,micromolar
x,1,Sertraline,0.5,micromolar
x,1,Quetiapine,3,micromolar
Repli,4
DMSOvol,0.4
minOrder,1
maxOrder,4
```

Figure S5: User-specified text file for COMBO-Pick in the context of the demonstrated CUSP9v4 case study. Instructions on how to produce such a specification file for COMBO-Pick are provided with the executable file, which can be downloaded. See "Availability of data and materials" section in the paper.

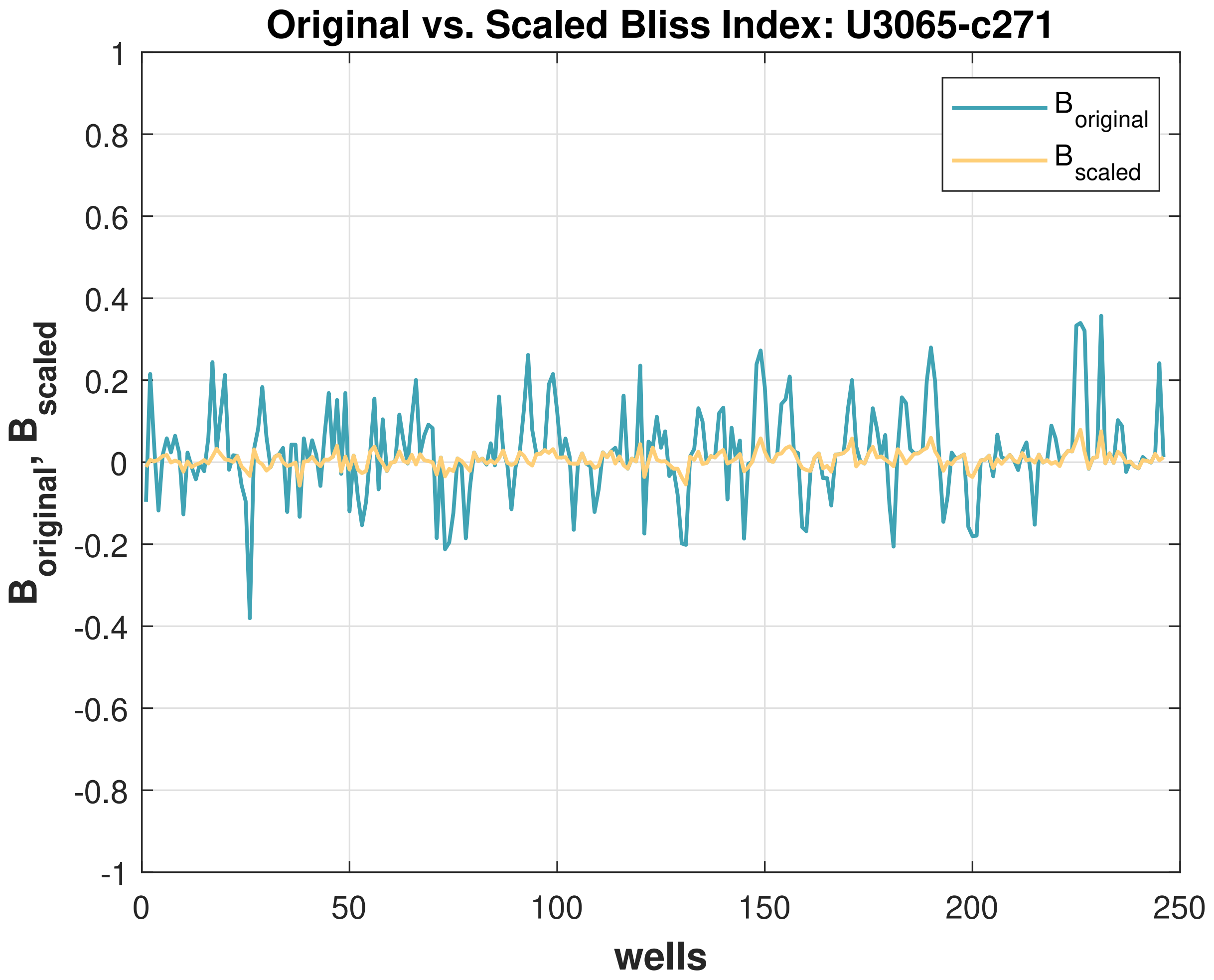

Figure S6: Conventional and scaled Bliss analyses for the semi-exhaustive experiment of the CUSP9v4 case study. The Bliss scores (y-axis) were calculated for all 246 drug combination wells (x-axis), which are shown in ascending combination order: wells no. 1-36 $\longmapsto$ 2-order combinations; wells no. 37-120 $\longmapsto$ 3-order combinations; wells no. 121-246 $\longmapsto$ 4-order combinations.

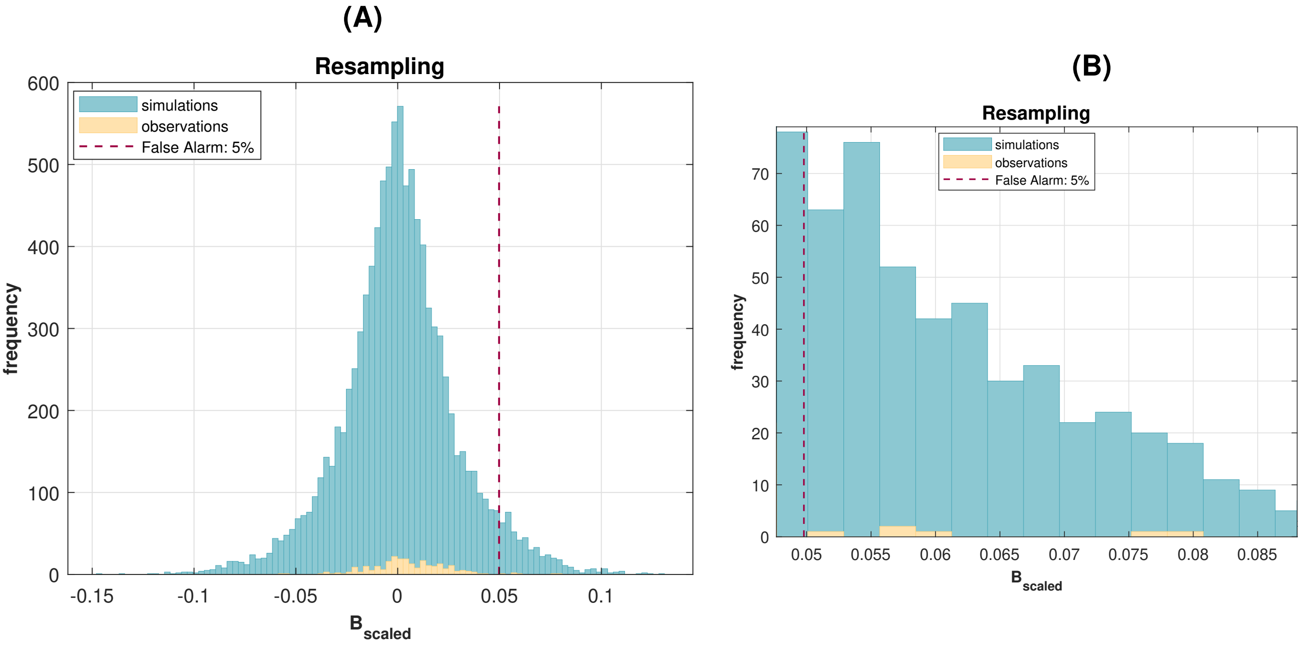

Figure S7: **(A)** Statistical analysis for the scaled Bliss synergy analysis using resampling and performing 10000 simulations when $B_{scaled} = 0$ (i.e., under the null hypothesis of Bliss independence); **(B)** Six detections were made at the 5% false alarm of the sampling distribution (zooming on in the histogram region from **(A)** that lies above the 5% false alarm threshold. The results are included in Table ST1 below.

**Drug Combinations**	$\mathbf{B_{scaled}}$	$\mathbf{B_{original}}$	$\mathbf{S_{observed}}$
(Aur, Cel, Itr, Ser)	0.0527	0.3331	0.8102
(Apr, Cel, Itr, Que)	0.0579	0.2005	0.7939
(Apr, Aur, Cel, Ser)	0.0581	0.2724	0.8201
(Min, Aur, Itr, Que)	0.0595	0.2797	0.8042
(Aur, Itr, Ser, Que)	0.0752	0.3571	0.7132
(Aur, Cel, Itr, Que)	0.0789	0.3395	0.8149

Table ST1: The six drug combinations of the CUSP9v4 case study that were identified as synergistic (according to Bliss) at the 5% false alarm level of the sampling distribution (under the null hypothesis of Bliss independence). The resampling based statistical analysis (Fig. S7) based on 10000 simulations was performed using the scaled Bliss index values (Fig. S6). The drug combinations are sorted in ascending order with respect to the scaled Bliss index values, while the conventional/original Bliss index values along with the observed survival index values are also provided. It is obvious that high positive Bliss index values that are related to high observed survival index values were successfully suppressed by our rescaling method.

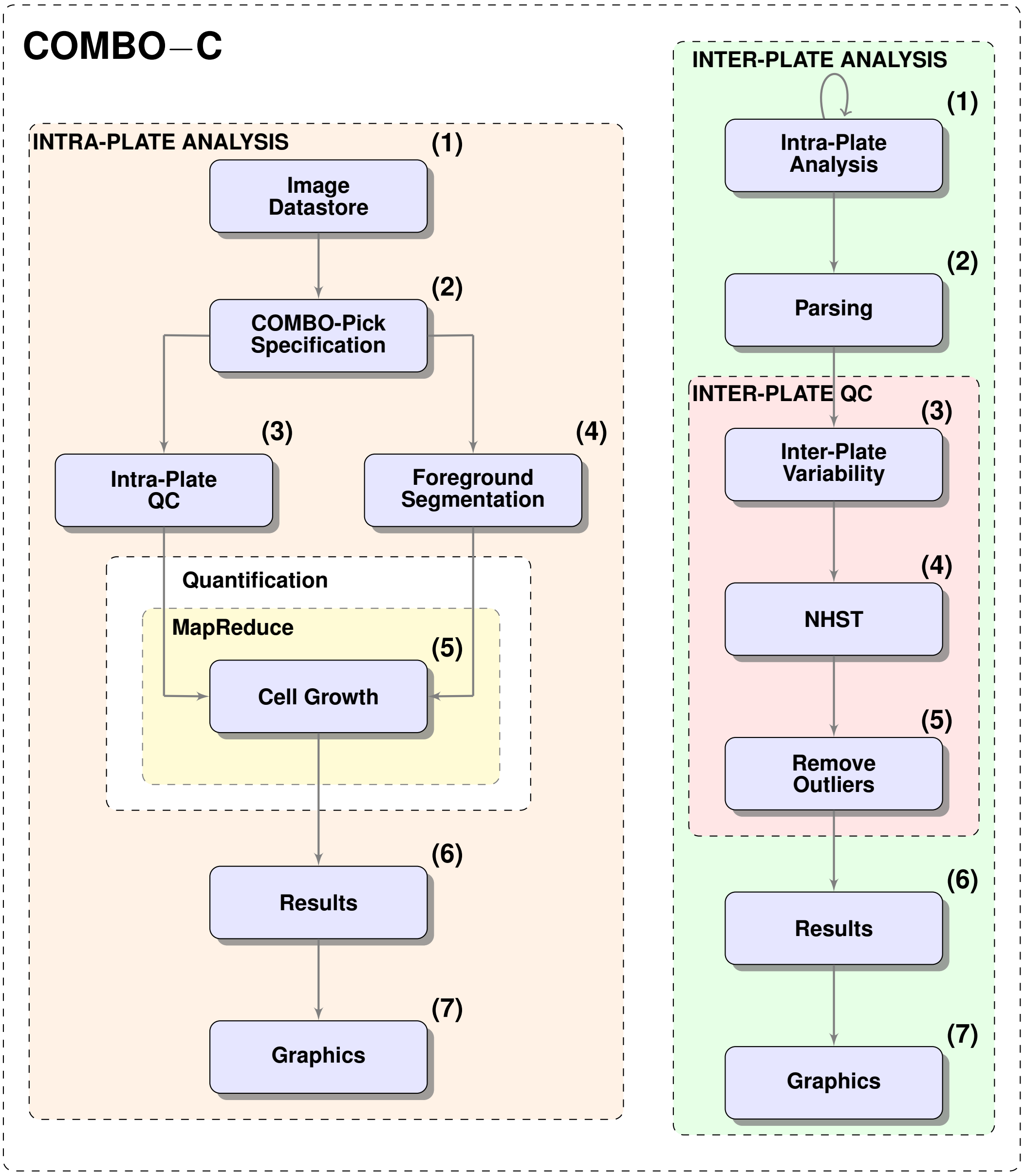

Figure S8: INTRA-PLATE ANALYSIS: (1) Image datastore selected by the user; **(2)** COMBO-Pick specification imported by the user; **(3)** MapReduce-based intra-plate quality control; **(4)** Foreground segmentation; **(5)** MapReduce-based quantification of changes in cell growth relative to the first recorded time point; **(6)** Table (CSV) with results; **(7)** Temporal graphics (EPS, PDF). **INTER-PLATE ANALYSIS: (1)** Intra-plate analysis employed separately for all replicates; **(2)** Results from **(1)** gathered and parsed; **(3)** Inter-plate variability estimation; **(4)** Null hypothesis significance testing (NHST) based on resampling for outlier detection; **(5)** Detected inter-plate outliers are removed; **(6)** Table (CSV) with merged inter-plate replicate values; **(7)** Temporal graphics (EPS, PDF).

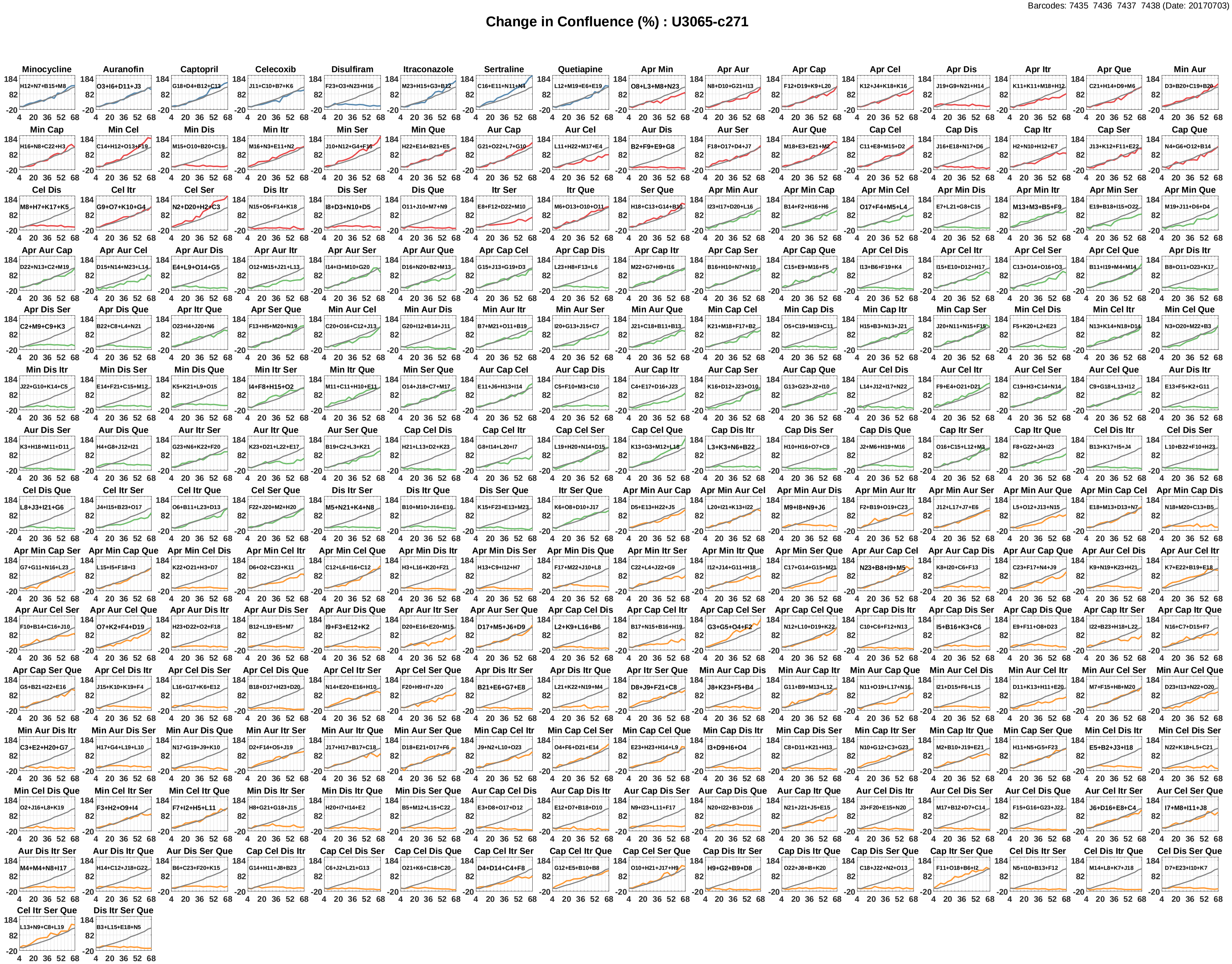

Figure S9: Growth curves for the sensitive GIC clone ($U3065 - c271$). The median values among the 4 replicates for a particular treatment are jointly shown together with corresponding values for untreated cells (gray curve). The x-axis corresponds to the recorded time in hours, starting at $4h$ and ending at $68h$ after drug addition, respectively. The y-axis shows the cell growth (%) with respect to $4h$. The corresponding well names are shown in each subplot.

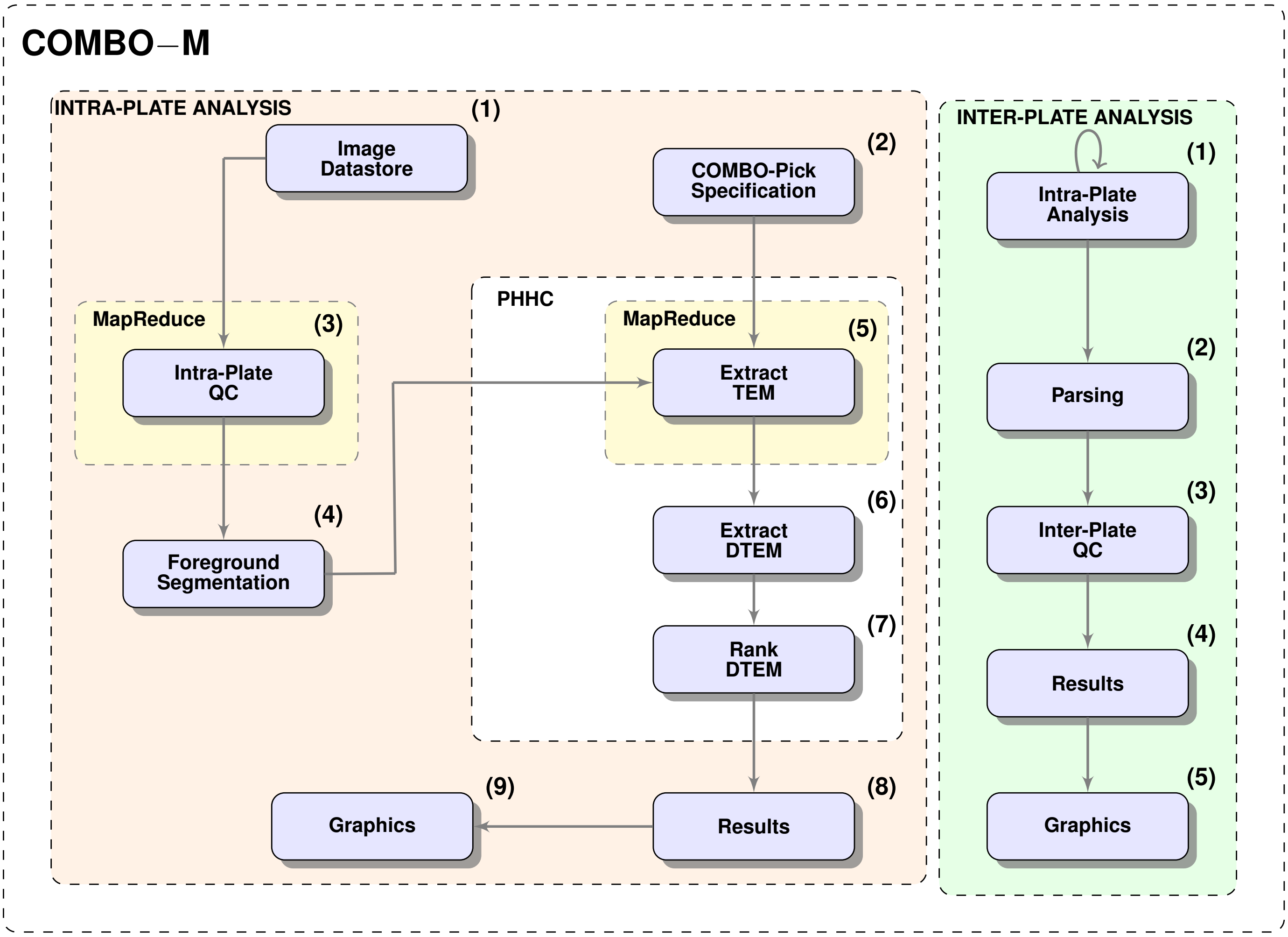

Figure S10: INTRA-PLATE ANALYSIS: (1) Image datastore selected by the user; **(2)** COMBO-Pick specification imported by the user; **(3)** MapReduce-based intra-plate QC; **(4)** Foreground segmentation; **(5)-(7)** MapReduce-based quantification of DTEM using the pixel histogram hierarchy and comparison (PHHC) algorithm; **(8)** Table (CSV) with results; **(9)** Temporal graphics or heatmaps (EPS, PDF). **INTER-PLATE QUALITY ANALYSIS: (1)** Intra-plate analysis employed separately for all replicates; **(2)** Results from **(1)** gathered and parsed; **(3)** Outlier removal based on the Inter-Plate QC as performed by COMBO-C; **(4)** Table (CSV) with merged inter-plate replicate values; **(5)** Temporal graphics or heat maps (EPS, PDF).

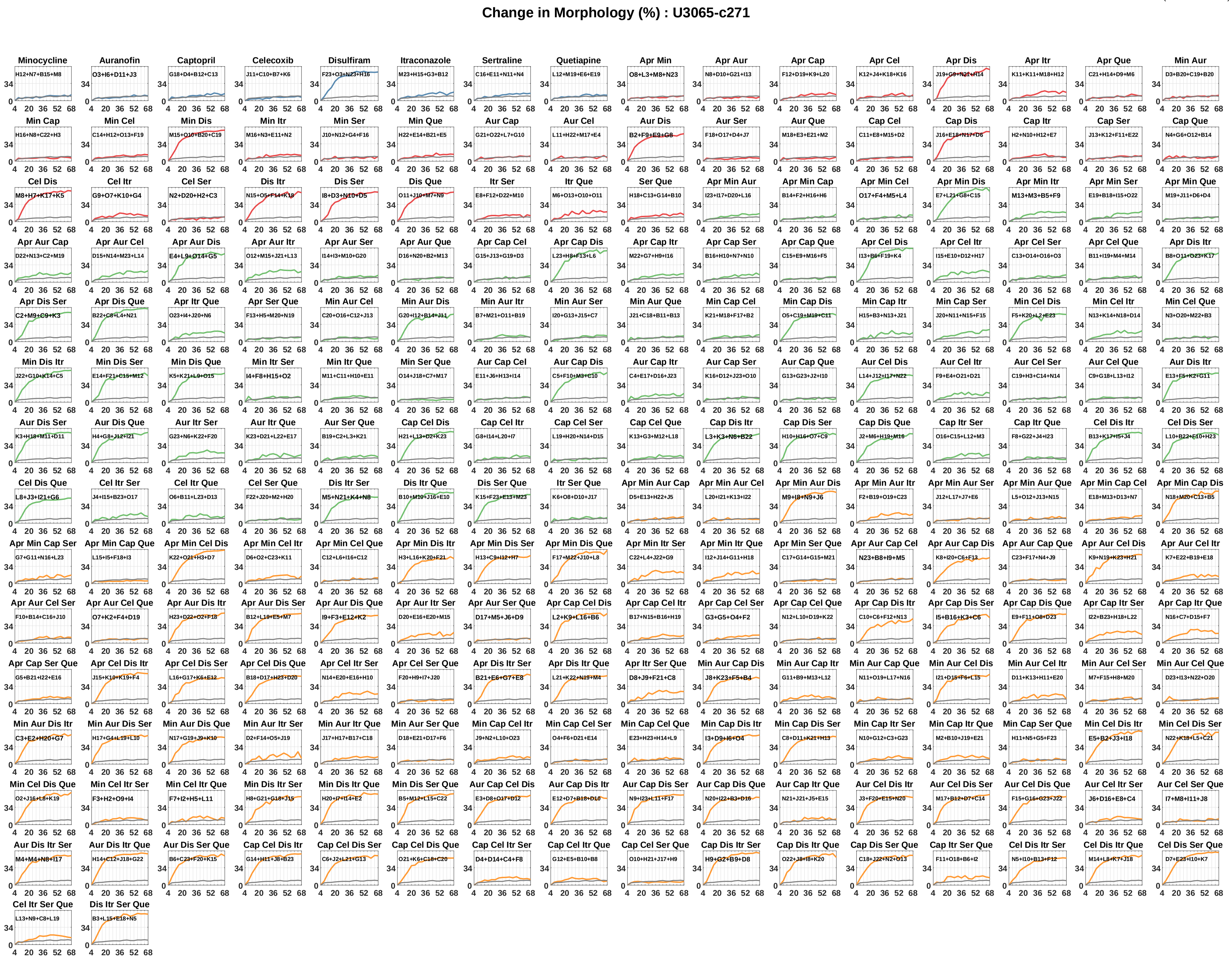

Figure S11: Temporal curves showing the changes in morphology for the sensitive GIC clone ($U3065-c271$). The median values among the 4 replicates for a particular treatment are jointly shown together with corresponding values for untreated cells (gray curve). The x-axis corresponds to the recorded time in hours, starting at $4h$ and ending at $68h$ after drug addition, respectively. The subplots are sorted in ascending order of combination order. The y-axis shows the change in morphology (%) with respect to $4h$. The corresponding well names are shown in each subplot.

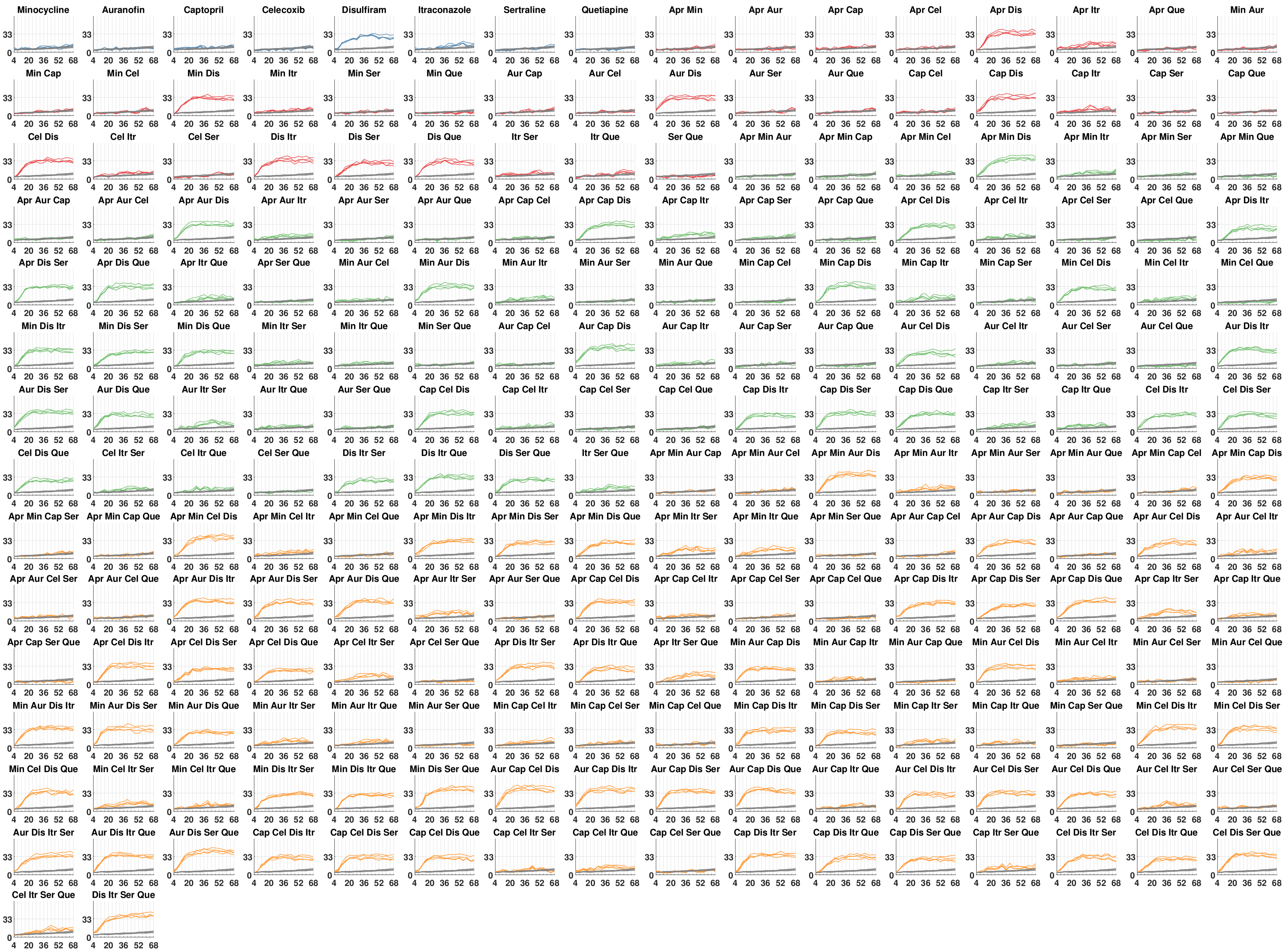

Figure S12: Temporal curves showing the number of apoptotic-like cells for the sensitive GIC clone ($U3065 - c271$) by employing four different but relatively similar prototypical objects (Fig. 6, main text). For all different prototypes, the median values among the 4 replicates for a particular treatment are jointly shown together with corresponding values for untreated cells (gray curve). The x-axis corresponds to the recorded time in hours, starting at $4h$ and ending at $68h$ after drug addition, respectively. The subplots are sorted in ascending order of combination order. The y-axis shows the detected apoptotic-like cell counts. The corresponding well names are shown in each subplot.

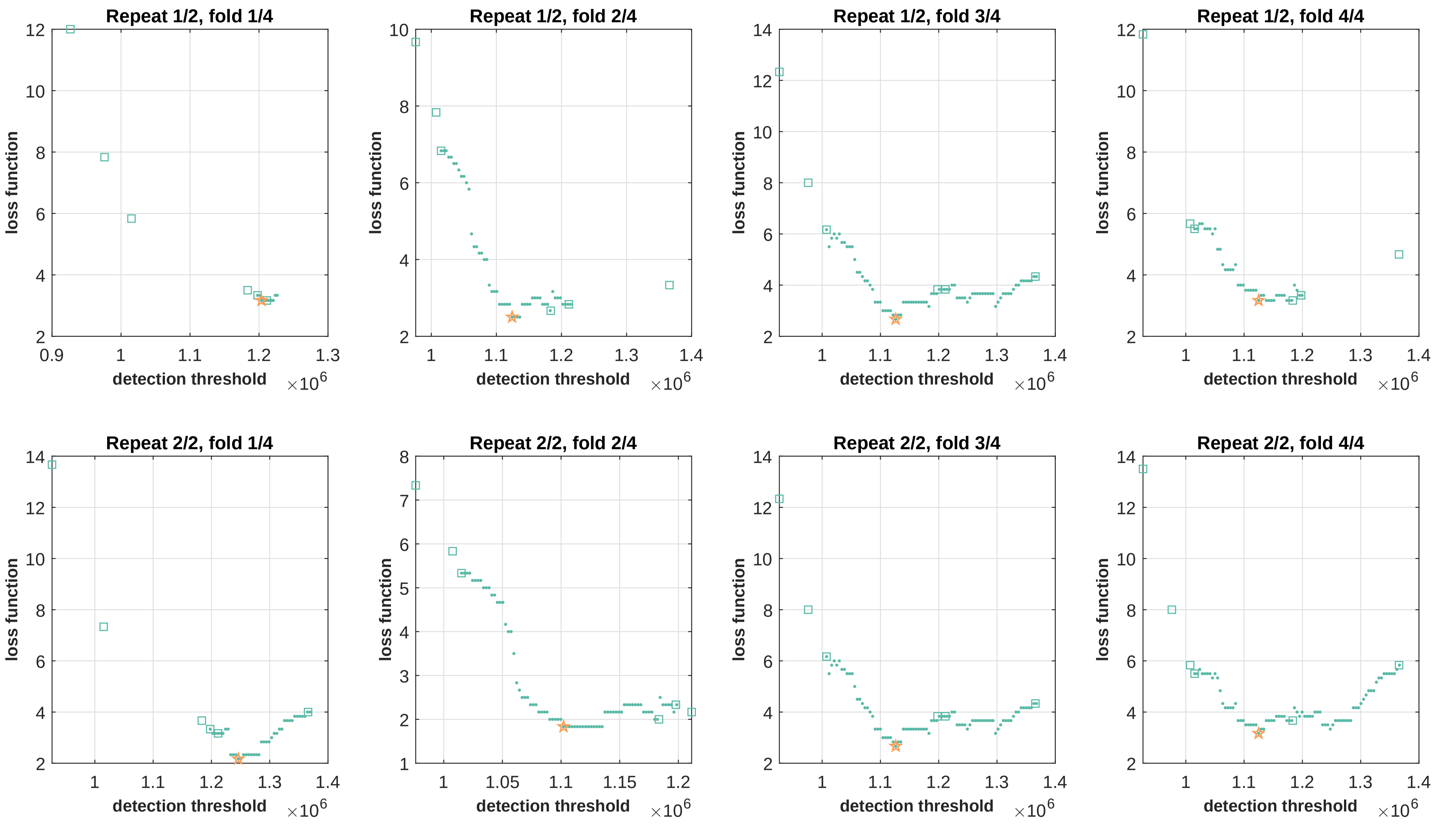

Figure S13: The interval optimization search method is illustrated for every partition of the employed cross validation procedure. The two rows correspond to the two independent repeats, whereas the four columns to the four folds. The starting points are shown as green squares, while the exhaustive search inside an appropriate interval is indicated with green dots. The optimal detection threshold found is shown as an orange star inside the corresponding optimization search interval. The median value of all local optima (orange stars) corresponds to the final optimal value used as the detection threshold.

Number of Apoptotic-like Objects : U3065-c271

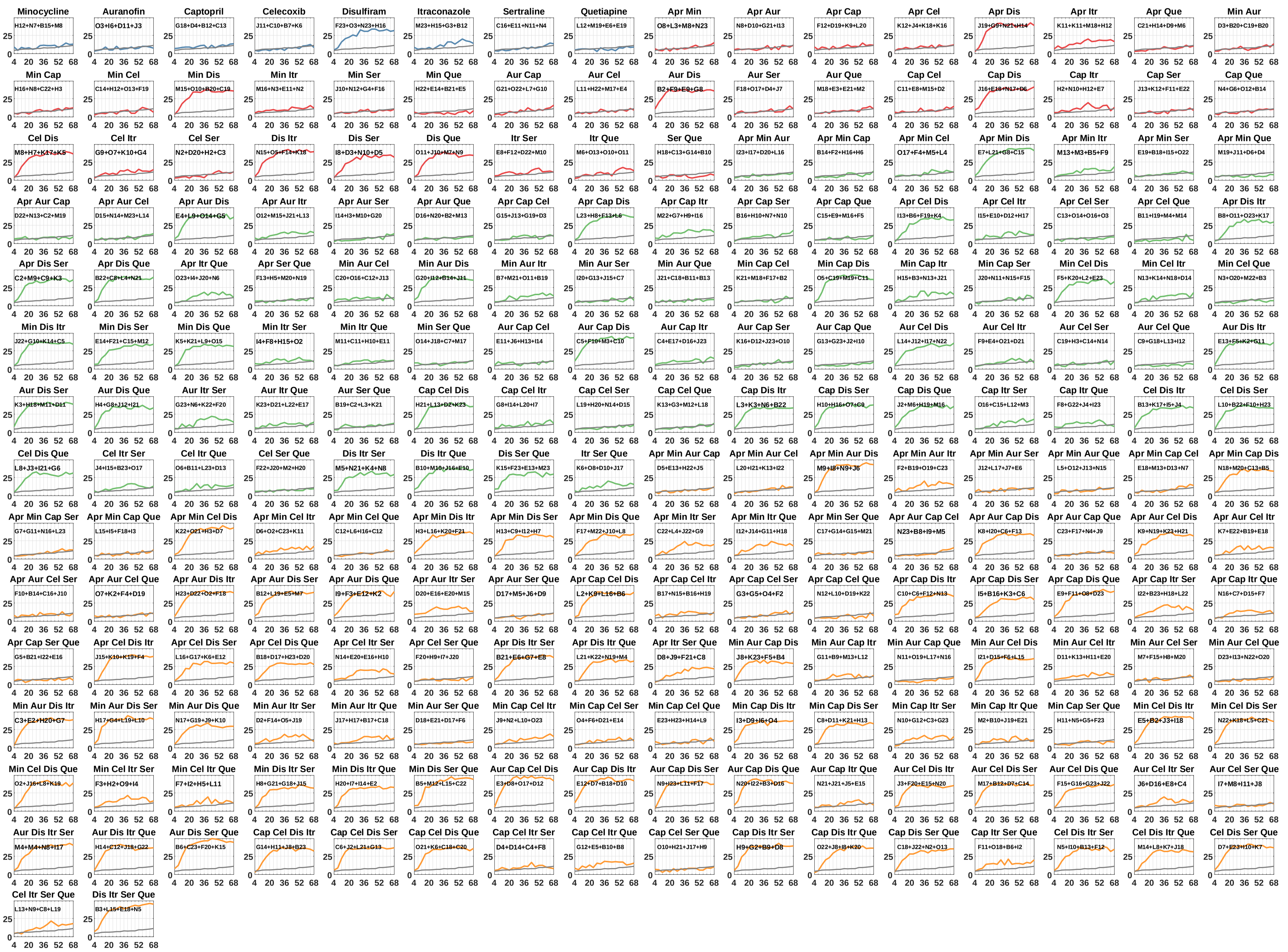

Figure S14: Temporal curves showing the number of apoptotic-like cells for the sensitive GIC clone ($U3065-c271$). The median values among the 4 replicates for a particular treatment are jointly shown together with corresponding values for untreated cells (gray curve). The x-axis corresponds to the recorded time in hours, starting at $4h$ and ending at $68h$ after drug addition, respectively. The subplots are sorted in ascending order of combination order. The y-axis shows the detected apoptotic-like cell counts. The corresponding well names are shown in each subplot.

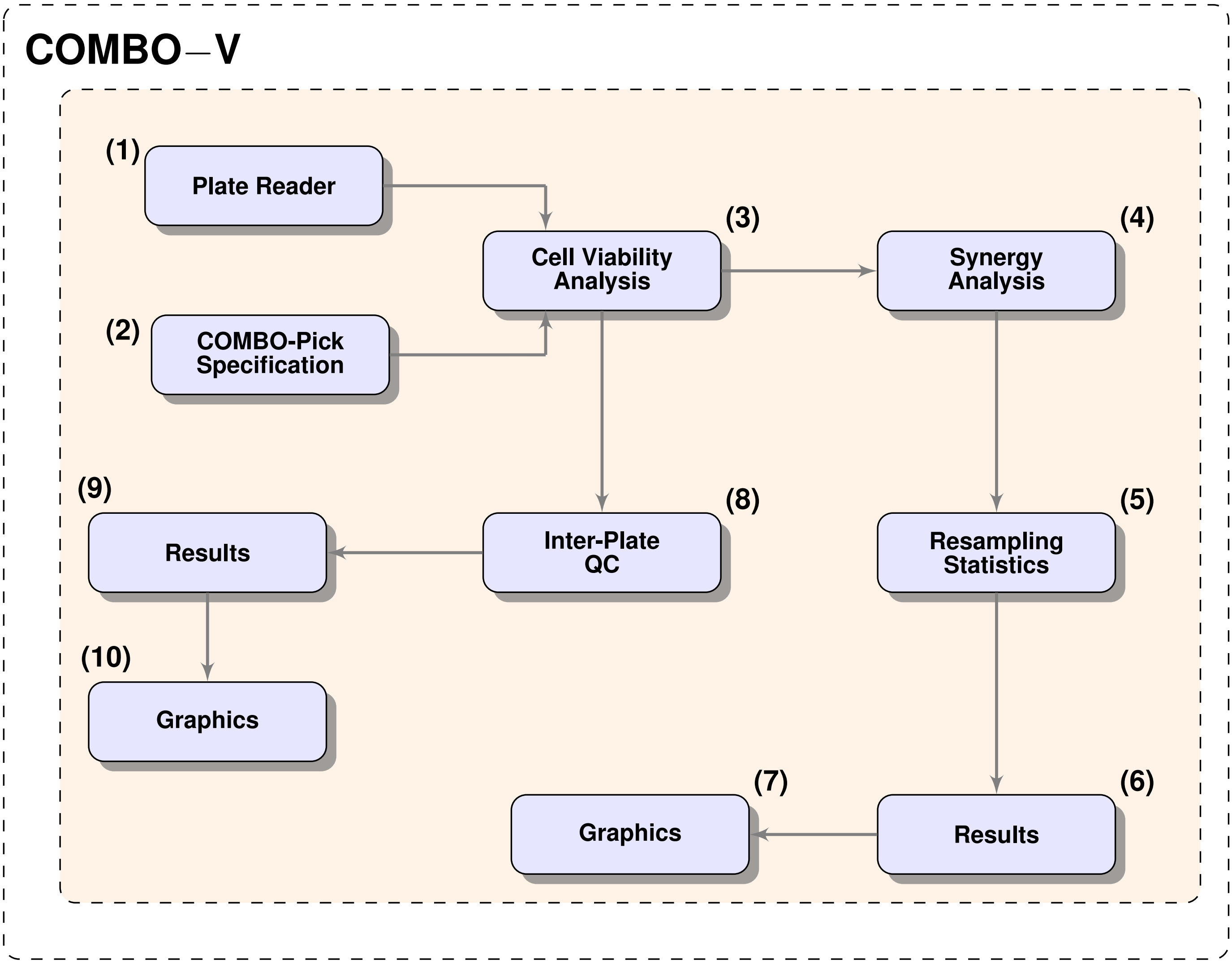

Figure S15: **(1)**-**(2)** Plate reader and COMBO-Pick specification(s) imported by the user; **(3)** Cell viability analysis; **(4)** Conventional and scaled Bliss and therapeutic synergy analyses; **(5)** Resampling statistics for **(4)**; **(6)** Table (CSV) with results; **(7)** Scatter plots or heat maps (EPS, PDF); **(8)** Outliers are excluded based on the inter-plate QC performed by COMBO-C; **(9)** Table (CSV) with results after **(8)**; **(10)** Scatter plots or heat maps (EPS, PDF) after **(8)**.

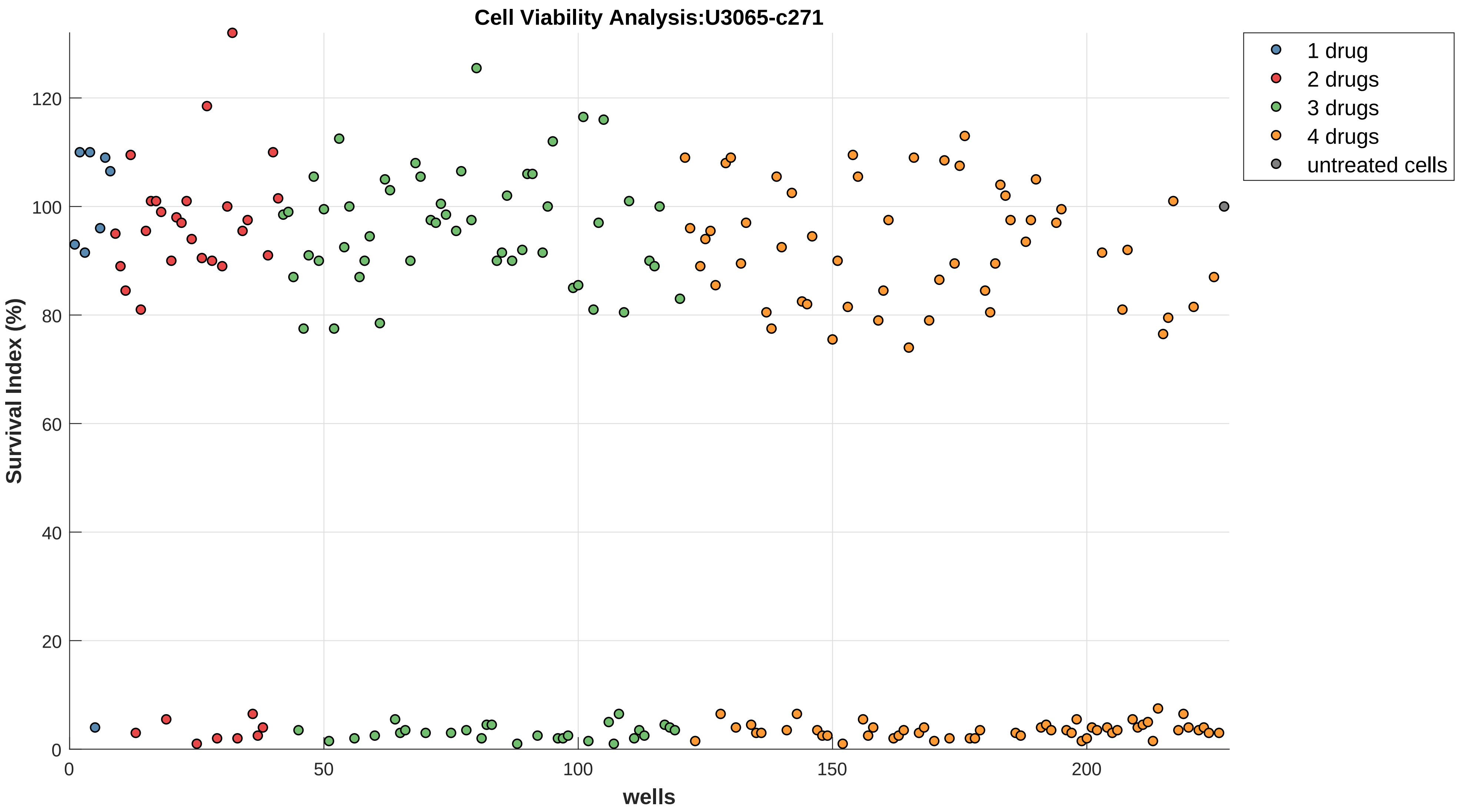

Figure S16: Cell viability for the sensitive GIC clone ($U3065-c271$) after $68h$ drug exposure. The median values among the 4 replicates for a particular treatment are shown together with the global median of all untreated wells (gray circle at the end). The x-axis corresponds to the experimental wells. The y-axis shows the survival index (%).

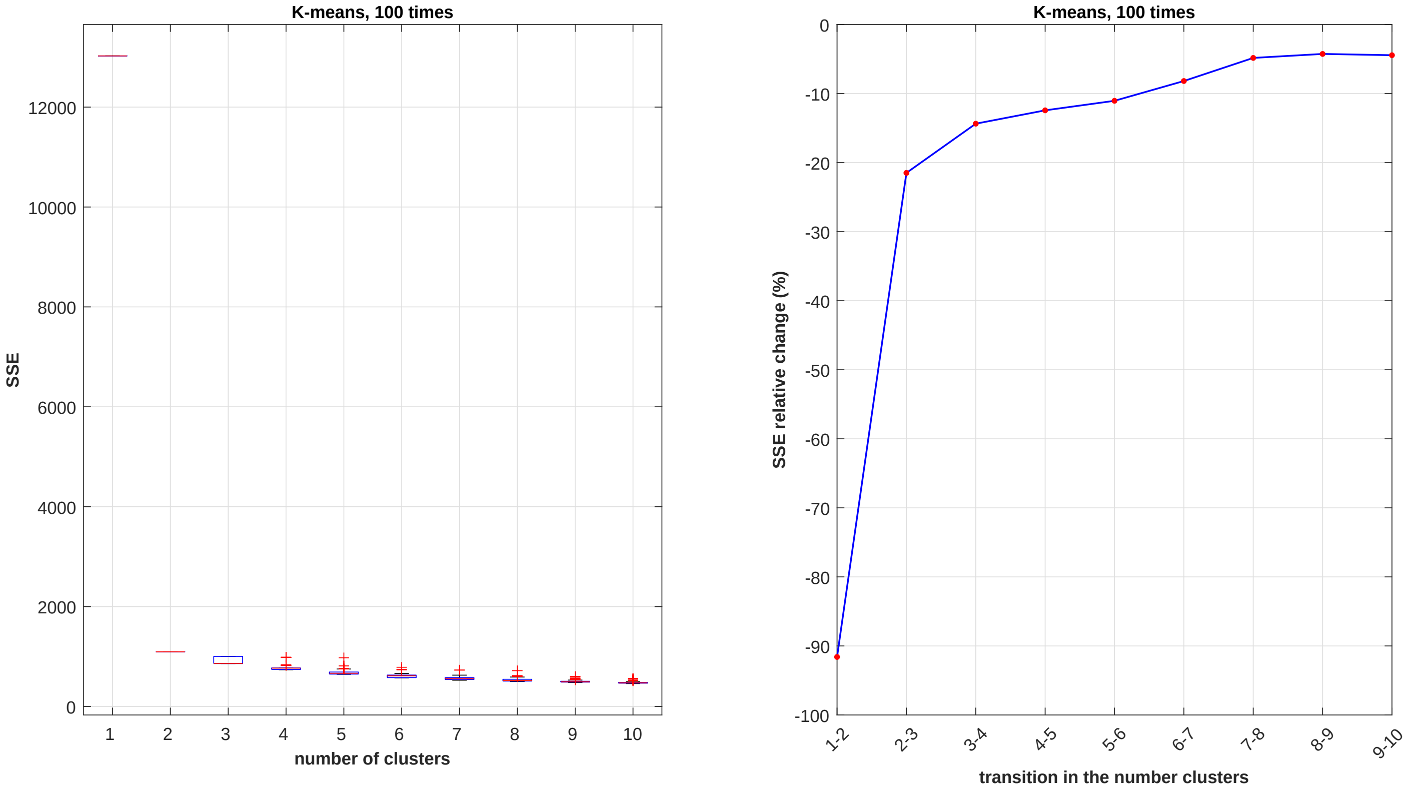

Figure S17: Results of employing K-means clustering in order to group the drug combination effects with respect to their temporal phenotypic behaviors. The clustering procedure was performed 100 times independently for $K = \{1, 2, \cdots 10\}$. The distributions of the corresponding sum of squared errors (SSE) are shown as box plots on the left side of the figure. The final selection of K was determined by calculating the relative change in SSE when transitioning between consecutive values of K, as illustrated on the right subplot. The smallest K that resulted in SSE drop bigger than 20% compared to the previous value $K - 1$, was selected. Here, $K = 2$ was selected.

(a) $K = 2$ was used for sub-clustering of cluster #1

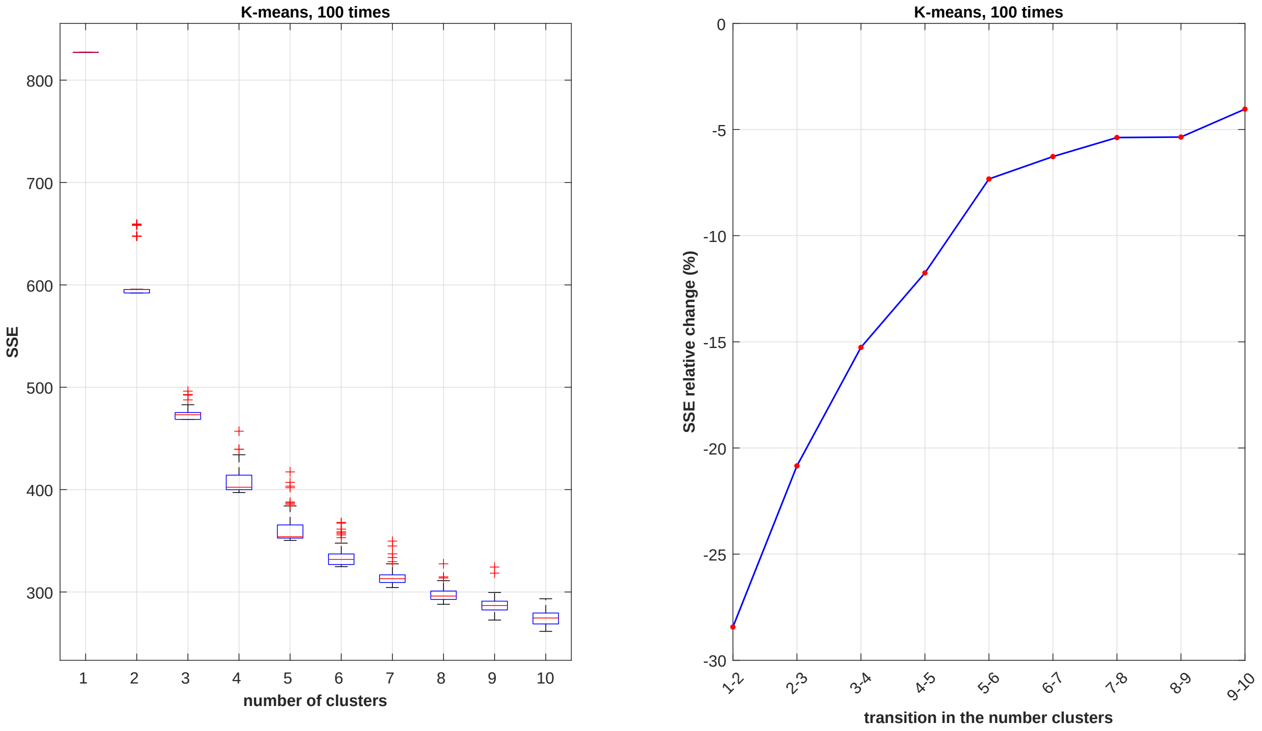

(b) $K = 2$ was used for sub-clustering of cluster #2

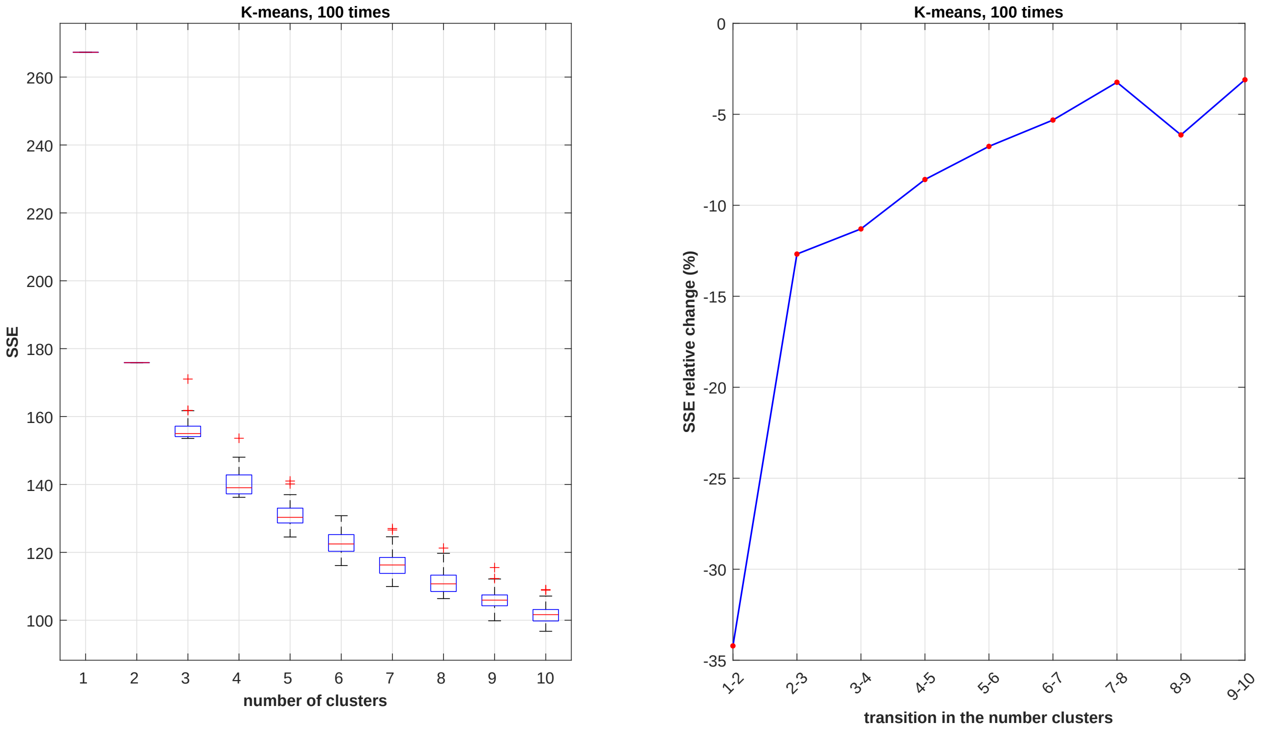

Figure S18: The choice of K for K-means clustering in the second hierarchical level, shown here, was performed similarly to the first hierarchical level, as described in fig. S17.

Appendices

A Algorithms

Algorithm SA1 Tuning of the matched filter threshold

Inputs

$\widetilde{f}$: loss function to be minimized
$\boldsymbol{\tau}_{init}$: n-dimensional vector with the initial detection thresholds
m: number of equidistant values in the initial interval (user defined)
Δ: factor for the search of right and left extremes if not any(user defined)

Output

$\boldsymbol{\tau}^*$: optimal threshold value

function MFThresholdingTuning($\boldsymbol{\tau}_{init}$, $\widetilde{\mathbf{f}}_{init}$, m, Δ)
  $\{\tau_{low}, \tau_{\text{2nd low}}, \cdots, \tau_{\text{2nd high}}, \tau_{high}\} \leftarrow \text{sort}\{\boldsymbol{\tau}_{init}\}, \quad \tau_{step} \leftarrow \frac{\tau_{high}-\tau_{low}}{m}$
  $\tau^*_{init} \leftarrow \min_{\{\tau \in \boldsymbol{\tau}_{init}\}} \widetilde{f}(\tau)$
  if τ^*_{init} is a vector **then**
    $\tau^*_{init,L} \leftarrow min\{\boldsymbol{\tau}^*_{init}\}, \; \tau^*_{init,R} \leftarrow max\{\boldsymbol{\tau}^*_{init}\}$
    $\tau_L \leftarrow max\{\tau\}, \; \{\tau \in \tau_{init} : \tau < \tau^*_{init,L}, \; \widetilde{f}(\tau) > \widetilde{f}(\tau^*_{init,L})\}$
    if $\tau_L \equiv \{\emptyset\}$ **then**
      $\tau_L \leftarrow$ findExtreme$(\widetilde{f}, \tau^*_{init,L}, -\Delta, \tau_{step})$
    end if
    $\tau_R \leftarrow min\{\tau\}, \; \{\tau \in \tau_{init} : \tau > \tau^*_{init,R}, \; \widetilde{f}(\tau) > \widetilde{f}(\tau^*_{init,R})\}$
    if $\tau_R \equiv \{\emptyset\}$ **then**
      $\tau_R \leftarrow$ findExtreme$(\widetilde{f}, \tau^*_{init,R}, \Delta, \tau_{step})$
    end if
  else
    if $\widetilde{f}(\tau^*_{init}) \leftarrow 0$ **then**
      $\tau^* \leftarrow \tau^*_{init}$
    else
      $\tau_L \leftarrow \tau, \; \{\tau \in \tau_{init} : \tau < \tau^*_{init}, \; \widetilde{f}(\tau) > \widetilde{f}(\tau^*_{init})\}$
      if $\tau_L \equiv \{\emptyset\}$ **then**
        $\tau_L \leftarrow$ findExtreme$(\widetilde{f}, \tau^*_{init}, -\Delta, \tau_{step})$
      end if
      $\tau_R \leftarrow \tau, \; \{\tau \in \tau_{init} : \tau > \tau^*_{init}, \; \widetilde{f}(\tau) > \widetilde{f}(\tau^*_{init})\}$
      if $\tau_R \equiv \{\emptyset\}$ **then**
        $\tau_R \leftarrow$ findExtreme$(\widetilde{f}, \tau^*_{init}, \Delta, \tau_{step})$
      end if
    end if
  end if
  $\tau_{cur} \leftarrow \tau_L, \; \widetilde{f}_{opt} \leftarrow \{\widetilde{f}(\tau_{cur})\}, \; \tau_{opt} \leftarrow \{\tau_{cur}\}$
  while $\tau_{cur} \leq \tau_R$ **do**
    $\tau_{cur} \leftarrow \tau_{cur} + \tau_{step}, \; \tau_{opt} \leftarrow \{\tau_{opt}, \tau_{cur}\}, \; \widetilde{f}_{opt} \leftarrow \{\widetilde{f}_{opt}, \widetilde{f}(\tau_{cur})\}$
  end while
  $\tau^* \leftarrow \min_{\tau \in \tau_{opt}} \widetilde{f}(\tau)$
  return τ^*
end function

Algorithm SA2 Find left and right extreme points for Algorithm SA1

Inputs

$\widetilde{f}$: loss function to be minimized

τ_{opt}: current local minima

Δ: factor for the search of extremes (user defined)

Output

τ_{step}: step for the search as determined by Algorithm SA1

function FINDEXTREME($\widetilde{f}$, τ_{opt}, Δ, τ_{step})
    $\tau \leftarrow \tau_{opt}$
    while $\widetilde{f}(\tau) \leq \widetilde{f}(\tau_{opt})$ **do**
        $\tau \leftarrow \tau + \Delta \cdot \tau_{step}$
    end while
    return τ
end function
